# Structural and Functional Characterization of the *N*‑Prenyltransferase DsKabA in Algal Neurotoxin Biosynthesis

**DOI:** 10.1021/acs.jafc.6c12021

**Published:** 2026-10-28

**Authors:** Wei-Jia Huang, Sheng-Chia Chen, I-Weh Chien, Chia-Hsin Lin, Jia-Yi Zhou, Bo-Yan Guo, San-Chi Chang, Sheng-Wei Lin, Chun-Hua Hsu

**Affiliations:** † Department of Agricultural Chemistry, 33561 National Taiwan University, Taipei 106319, Taiwan; ‡ Institute of Biochemical Sciences, National Taiwan University, Taipei 106319, Taiwan; § Department of Seafood Science, National Kaohsiung University of Science and Technology, Kaohsiung 811213, Taiwan; ∥ Department of Microbiology, Soochow University, Taipei 111002, Taiwan; ⊥ Genome and Systems Biology Degree Program, National Taiwan University and Academia Sinica, Taipei 106319, Taiwan; # Center for Computational and Systems Biology, National Taiwan University, Taipei 106319, Taiwan

**Keywords:** kainic acid, *N*-prenyltransferase, crystal structure, biosynthesis

## Abstract

Kainic acid (KA) is a neuroactive natural product from the red alga *Digenea simplex*. Its biosynthesis begins with DsKabA, an *N*-prenyltransferase that converts l-glutamate and dimethylallyl pyrophosphate into prekainic acid, the first committed intermediate. Here, we combined LC-MS activity assays, X-ray crystallography, SAXS, molecular docking, ITC, MD simulations, and mutagenesis to characterize DsKabA. Product analysis confirmed prekainic acid formation, and kinetic measurements yielded apparent *K*m values of 1.03 ± 0.16 μM for DMAPP and 52.25 ± 1.61 mM for l-Glu. The 2.85 Å apo structure revealed open and closed conformations involving the α7-loop-α8 region near the active site. Docking, ITC, and mutagenesis support Mg^2+^-dependent donor binding, ordered l-Glu association, and roles for Y403, H406, E407, M114, and M118 in catalysis and prenyl-donor recognition. These findings provide structural and mechanistic insight into the first committed step of KA biosynthesis.

## Graphical abstract

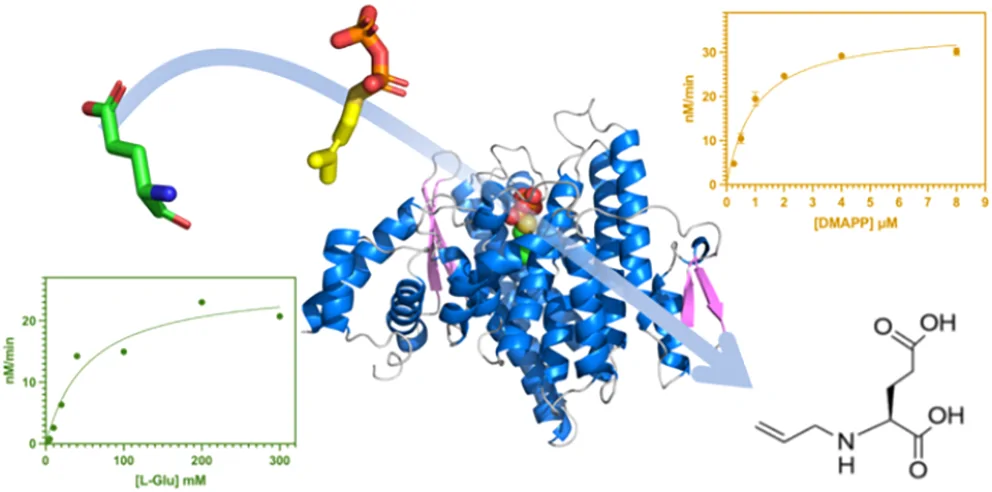

## Introduction

Kainic acid (KA) and its structural analogs are nonproteinogenic excitatory amino acids with significant neurotoxic and pharmacological properties. Originally isolated from the red alga *Digenea simplex*, KA has been widely employed in neuroscience as a selective agonist for kainate receptors, facilitating studies of synaptic transmission, neurodegeneration, and epilepsy.^1,2^ Beyond its neurological relevance, KA and related compounds exhibit potent anthelmintic and insecticidal activities, rendering them promising candidates for agricultural applications.^3^ KA exerts its biological effects through activation of ionotropic glutamate receptors (iGluRs), particularly kainate receptors (KARs), leading to excessive neuronal excitation and excitotoxicity.^4–6^ Accordingly, KA has served as a model compound in research on epilepsy and neurodegenerative disorders such as Alzheimer’s, Parkinson’s, and Huntington’s disease.^2,7–9^

Although widely utilized as a neuropharmacological tool, the biosynthetic origin of KA remained elusive until recent genomic studies revealed its biosynthetic gene cluster (BGC) in *D. simplex*.^10^ This BGC encodes two key enzymes: DsKabA, an *N*-prenyltransferase that catalyzes the transfer of a prenyl group from dimethylallyl pyrophosphate (DMAPP) to l-glutamate (l-Glu) to yield prekainic acid (PKA), and DsKabC, an α-ketoglutarate-dependent dioxygenase that converts PKA into KA (Scheme 1). Notably, the prenylation step catalyzed by DsKabA represents the first committed transformation in KA biosynthesis and establishes its core scaffold.^7^

**Scheme 1:**
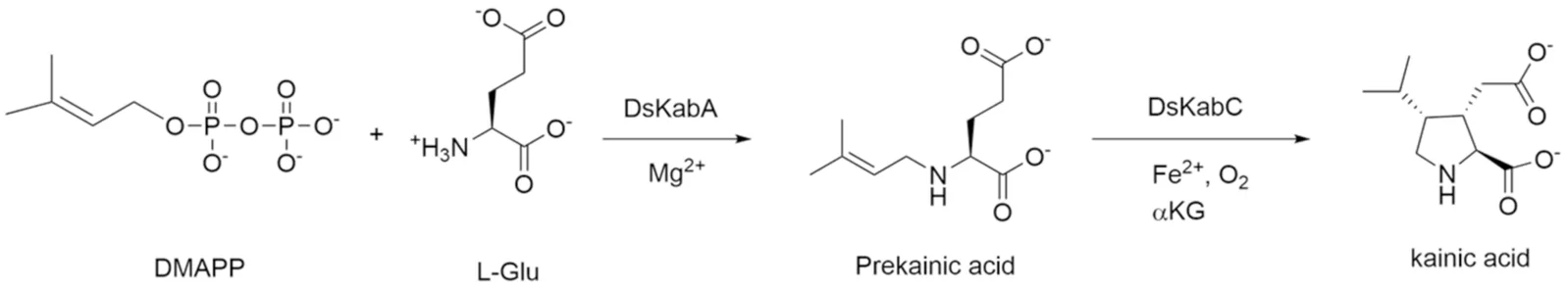
Biosynthetic Pathway of Kainic Acid^a^

a DsKabA catalyzes the Mg^2+^-dependent N-prenylation of l-glutamate (l-Glu) using dimethylallyl pyrophosphate (DMAPP) to generate prekainic acid (PKA). PKA is subsequently converted to kainic acid by the Fe^2+^/α-ketoglutarate-dependent enzyme DsKabC in the presence of O_2_.

*N*-prenyltransferases are essential catalysts in specialized metabolism, introducing prenyl moieties that enhance membrane affinity, stability, and bioactivity of natural products.^11–13^ Among them, enzymes with terpene cyclase-like folds form a unique structural subclass whose conformational dynamics and catalytic principles remain poorly understood. A recent structural study of DabA, a homologous *N*-prenyltransferase from *Pseudonitzschia*, revealed substrate- and product-bound analog structures.^7^ Although this study established the structural basis for DabA/DsKabA-type *N*-prenyltransferases, the ligand-free apo conformation and local conformational changes associated with substrate access and product release remain less well characterized.

In this study, we present biochemical and structural analyses of DsKabA to address these outstanding questions. We determined the crystal structure of DsKabA in its ligand-free state and captured two distinct conformations within the same asymmetric unit: a closed form that resembles the ligand-bound state of DabA, and an open form likely representing substrate entry or product release. These conformational states, supported by solution-phase small-angle X-ray scattering (SAXS) and molecular dynamics simulations, suggest a gating mechanism that regulates substrate access. Furthermore, isothermal titration calorimetry (ITC) and structure-guided mutagenesis revealed stepwise substrate binding and identified key catalytic residues. Together, our study extends the current mechanistic framework for DabA/DsKabA-type *N*-prenyltransferases by providing structural and biophysical insight into the apo state, conformational flexibility, and active-site determinants of DsKabA.

## Materials and Methods

### Materials

The *Escherichia coli* strain BL21 (DE3) and the pET-30a plasmid (Novagen, Madison, WI, USA) were used for protein expression. Restriction endonucleases and T4 DNA ligase were purchased from Thermo Fisher Scientific (Hudson, NH, USA). PrimeSTAR HS DNA Polymerase was purchased from Takara Bio (Otsu, Japan). All other chemicals and reagents were purchased from Sigma-Aldrich Chemical Co. (St Louis, MO, USA).

### Protein Expression and Purification

The DNA sequence encoding DsKabA (Genbank accession number: QCC62379.1) was synthesized (MDbio, Taiwan) and inserted into the pUC57 plasmid. Subsequently, the gene fragment of DsKabA was inserted between the NdeI and XhoI sites of the pET-30a vector system (Novagen). Transformants were selected on Luria–Bertani (LB) medium containing 50 μg/mL kanamycin following overnight incubation at 37 °C.

The resulting plasmid with the inserted sequence was then transformed into *E. coli* BL21­(DE3). The cells were grown at 37 °C until reaching an OD_600_ of 1.0, with 50 μg/mL of kanamycin. The expression of the recombinant DsKabA with a His-tag at the C-terminus was induced in the cells by 0.1 mM isopropyl-β-D-thiogalactoside, followed by growth for 18 h at 16 °C. Cells were collected by centrifugation and resuspended in lysis buffer (20 mM Tris-HCl pH 8.0, 500 mM NaCl). After 20 min of sonication, the cell extract was clarified by centrifugation at 12,000 rpm for 20 min at 4 °C to remove debris. The clear supernatant was placed in an open column filled with Ni-NTA resin. The resin was washed with ten times the volume of lysis buffer containing 50 mM and 100 mM imidazole, respectively. The His-tagged DsKabA was eluted by lysis buffer containing 300 mM imidazole.

Site-directed mutants of DsKabA were generated using overlap-extension PCR with the primers listed in Table S1. The mutations were confirmed by sequencing. Recombinant proteins were expressed in *E. coli* BL21­(DE3) and purified following the same protocol used for the wild-type protein.

### Gel Filtration Chromatography

Gel filtration chromatography (Superdex 200 Increase 10/300 GL, GE Healthcare) using a ÄKTA pure FPLC system was used to further purify and analyze the oligomeric state of DsKabA. Chromatography was performed using 20 mM Tris-HCl buffer (pH 8.0) and 100 mM NaCl as the mobile phase, at a flow rate of 0.5 mL/min. The calibration curve was determined with gel-filtration molecular weight standards containing Thyroglobulin (670 kDa), γ-globulin (158.0 kDa), Ovalbumin (44.0 kDa), and Myoglobin (17.0 kDa).

### SEC-MALS Experiment

For molecular weight and quaternary structure determination, SEC-MALS analysis of DsKabA protein was performed. The column (Enrich Tm SEC. 70 10 × 300, Bio-Rad Laboratories, Santa Barbara, CA, USA) was used with a flow rate of 0.3 mL/min in the buffer system of 20 mM Tris pH 8.0, 100 mM NaCl, 5% glycerol and 25 °C. Detectors such as an ultraviolet–visible (UV) detector (QELS, Wyatt Technology, Santa Barbara, CA, USA), a static light-scattering detector (mini DAWN TREOS, Wyatt Technology, Santa Barbara, CA, USA), a quasi-elastic light-scattering detector (QELS, Wyatt Technology, Santa Barbara, CA, USA), and a refractive index detector (Optilab T-rEX, Wyatt Technology, Santa Barbara, CA, USA) all were aligned with the column. Bovine serum albumin (Sigma, A1900, Saint Louis, MO, USA) was used as a standard for calibration and optimization. The molecular weight was calculated by using ASTRA 6 with d*n*/d*c* value set to 0.185 mL g^–1^.

### Prekainic Acid Chemical Synthesis

The chemical synthesis method of PKA was modified based on a published reference.^10^ Briefly, l-Glu (0.147 g, 1.0 mmol) and NaOH (0.083 g, 2.1 mmol) were dissolved in 2.5 mL of water and stirred at room temperature until completely dissolved. Methanol (2.5 mL) and prenal (3-methyl-2-butenal; 0.135 mL, 1.4 mmol) were then added, followed by NaBH_4_ (0.048 g, 1.3 mmol). The reaction mixture was immediately placed in an ice bath and stirred for 40 min. The mixture was subsequently allowed to stand at room temperature for approximately 20 min and acidified with formic acid to approximately pH 4.

Methanol was removed under reduced pressure, and the resulting mixture was subjected to solid-phase extraction (SPE). The PKA-containing fraction was eluted with 30% acetonitrile containing 0.1% formic acid and further purified by preparative reversed-phase HPLC using a SunFire C18 OBD Prep Column (10 × 250 mm). Isocratic elution was performed with 30% acetonitrile containing 0.1% formic acid at a flow rate of 2 mL min^–1^, with UV absorbance monitored at 210 nm. The major PKA-containing fraction was collected and concentrated under reduced pressure, yielding 11.68 mg of PKA as a white powder.

The identity of the purified PKA was confirmed by ^1^H NMR and tandem mass spectrometry (MS/MS). ^1^H NMR spectra were recorded on a Bruker AVIII-800 NMR spectrometer (Bruker BioSpin, Rheinstetten, Germany). For MS/MS analysis, the sample was analyzed using a Waters SYNAPT HDMS Q-TOF mass spectrometer (Waters Corp., Manchester, UK) equipped with a nanospray ionization source and operated in positive ion mode. The NMR and MS/MS data were used together to confirm the identity of the synthesized PKA, which was subsequently used as an authentic standard for LC-MS quantification of enzymatic product formation.

### Circular Dichroism (CD) Spectroscopy

CD spectra were recorded using a Jasco J-815 spectropolarimeter (Jasco, Tokyo, Japan). Protein samples of wild-type DsKabA and variants were prepared at 20 μM in 20 mM sodium phosphate buffer at specified pH conditions. Far-UV spectra (190–260 nm) were measured at 25 °C using a 1 mm path-length quartz cuvette. Each spectrum was averaged from three scans collected at a scan rate of 20 nm/min with a 1 nm bandwidth. Buffer baselines were subtracted from sample spectra prior to analysis.

### LC-MS Analysis and Quantification of Enzymatic Products

Enzymatic products were analyzed using an Accela 1250 HPLC system (Thermo Fisher Scientific, Waltham, MA, USA) equipped with a PAL autosampler and coupled to a TSQ Vantage triple-stage quadrupole mass spectrometer (Thermo Fisher Scientific). Chromatographic separation was carried out on a Hypersil GOLD C18 column (2.1 × 100 mm, 1.9 μm; Thermo Fisher Scientific). The mobile phases consisted of 0.1% formic acid in water (solvent A) and 0.1% formic acid in acetonitrile (solvent B). The gradient program was as follows: 0–1.5 min, 2% B; 1.5–6 min, 2–98% B; 6–8 min, 98% B; 8–11 min, 98–2% B; and 11–15 min, 2% B. The flow rate was 0.5 mL min^–1^.

Mass spectrometric analysis was performed using an electrospray ionization (ESI) source operated in positive-ion mode. Target analytes were monitored by selected reaction monitoring (SRM). The spray voltage was set to 3250 V, and the collision energy was 10 eV for all target analytes. PKA was monitored using the SRM transition *m*/*z* 216.1 → 148.2, whereas the GPP-derived *N*-geranylated l-glutamate product (NGG) was monitored using the transition *m*/*z* 284.2 → 148.2. Data acquisition and processing were performed using Xcalibur 2.2 software.

For quantitative analysis of PKA formation, chemically synthesized and structurally verified PKA was used as an authentic standard. Matrix-matched calibration standards were processed and analyzed under the same conditions as the enzymatic reaction samples, and PKA concentrations were calculated from the integrated SRM peak areas using the corresponding calibration curve. Because an authentic standard for the GPP-derived product was not available, its formation was evaluated from the integrated SRM peak area and used only for relative activity comparisons.

### Enzyme Kinetic Analysis

Steady-state kinetic measurements were performed using freshly prepared DsKabA at a final concentration of 25 nM. Reactions were carried out in a total volume of 200 μL containing 20 mM Tris-HCl (pH 8.0), 300 mM NaCl, 5 mM MgCl_2_, and the indicated substrate concentrations. For determination of the apparent kinetic parameters for DMAPP, l-Glu was maintained at 100 mM while DMAPP concentration was varied. For determination of the apparent kinetic parameters for l-Glu, DMAPP was maintained at 50 μM while l-Glu concentration was varied. Reactions were initiated by addition of enzyme, incubated for 15 min under conditions within the linear range of product formation, and terminated by addition of an equal volume of 2% aqueous formic acid.

Initial velocities were calculated from the amount of PKA formed during the 15 min reaction period and fitted to the Michaelis–Menten equation by nonlinear regression. Because one substrate was maintained at a fixed concentration while the other was varied, the resulting kinetic parameters are reported as apparent *K*_m_ values under the specified assay conditions.

### Relative Activity Analysis of DsKabA Variants

Relative activities of wild-type DsKabA and selected variants were measured under the same assay conditions used for kinetic analysis. Reactions contained 25 nM enzyme, 100 mM l-Glu, and either 8 μM DMAPP or 60 μM GPP and were incubated for 15 min. For DMAPP-containing reactions, PKA formation was quantified using the synthetic PKA calibration curve. For GPP-containing reactions, formation of the corresponding *N*-geranylated l-glutamate product was evaluated from the integrated SRM peak area. For each prenyl donor, the activity of each variant was normalized to that of wild-type DsKabA measured under the same conditions, which was defined as 100%. All measurements were performed in triplicate, and the data are presented as mean ± SD.

### Crystallization and Data Collection

Initial protein crystallization trials were performed at 283 K by the sitting-drop vapor-diffusion method with commercial crystallization screen kits, 96-well Intelli-plates (Art Robbins Instruments). Each crystallization drop was prepared by mixing 1 μL DsKabA at 28 mg/mL with an equal volume of mother liquor, and the mixture was equilibrated against 300 μL reservoir solution. The crystals for data collection were grown in the condition of 0.1 M Bis-Tris pH 6.5, 20% w/v Polyethylene glycol monomethyl ether 5000 in 1 month at 283 K. For subsequent diffraction, the crystal was cryoprotected in mother liquor supplemented with 25% glycerol, and flash-frozen in liquid nitrogen at 100 K. The X-ray diffraction images were recorded in a 100-K nitrogen gas stream with use of Rayonix MX300HE CCD Area Detector on TLS-15A1 beamline (National Synchrotron Radiation Research Center, Taiwan). Individual frames consisted of a 1 ° oscillation angle measured for 10 s at a crystal-to-detector distance of 250 mm. The crystal belongs to tetragonal *P*4_1_2_1_2 with the following unit cell dimensions: *a* = 98.745, *b* = 98.745, *c* = 206.249. Intensity data were processed with HKL2000 software.^14^

### Structure Determination and Refinement

The crystal structure of DsKabA was determined by molecular replacement method with the Phaser^15^ of the PHENIX suite of programs^16^ using the DabA protein structure (PDB code: 6VKZ) as the searching model. The initial model was rebuilt interactively by inspecting the σ-weighted electron density maps with coefficients 2*mF*_o_–*DF*_*c*_ and *mF*_*o*_ *–DF*_*c*_ in COOT^17^ and then refinement with phenix.refine^18^ until *R*_work_ and *R*_free_ values converged. The geometric parameters of the final models were checked by using PROCHECK^19^ and the MolProbity program.^20^ The volume of active site was calculated by using CAVER.^21^ Molecular visualizations were generated with PyMOL (The PyMOL Molecular Graphics System, version 1.7, Schrödinger, LLC).

### Small-angle X-ray Scattering (SAXS)

SAXS was performed to analyze the shape and quaternary structure of DsKabA. SAXS measurements were conducted at the TPS-13A BioSAXS beamline (National Synchrotron Radiation Research Center, Taiwan). The purified DsKabA was prepared in the Tris-buffer (20 mM Tris HCl pH8, 100 mM NaCl) solution and further exposed X-rays with an online high-performance liquid chromatography (HPLC) system. The concentration of sample was 10 mg/mL. Total 119 SAXS profiles were then collected with a detecting system comprising two detectors, Eiger X 9 M and Eiger X 1 M. Protein samples were placed in a quartz capillary with a diameter of 2.0 mm using an exposure time of 1 s/frame. With an X-ray beam energy of 15.0 keV and a sample-to-detector distances of 2300.26 mm, the data covered a wide scattering vector q-range from 0.006 Å^–1^ to 1.8 Å^–1^ (q is the momentum transfer vector and is defined as q = (4π/λ) sin θ, where 2θ is the scattering angle, and λ is the wavelength of X-rays). The analysis of experimental data (buffer subtraction, averaging, merging, gyration radius calculation, Guinier analyses, p­(r) construction, bead modeling, molecular model validation) were made using the ATSAS program.^22^ The experimental SAXS data of DsKabA was fitted to crystal structure by the CRYSOL program.^23^ The envelope model was constructed by the DAMMIF program.^24^ The envelope diagram fitted crystal structure was produced by chimeraX.^25^

### Molecular Dynamics Simulation

All MD simulations were performed using GROMACS 2020.6.^26^ The CHARMM36m all-atom force field was employed for protein parametrization,^27^ and explicit solvation was modeled using the TIP3P water model.^28^ Three systems were constructed: (i) the apo form of DsKabA, (ii) the DMAPP-bound complex, and (iii) the ternary complex containing both l-Glu and DMAPP docked into the active site.

Each system was solvated in a dodecahedral box with a 12 Å buffer between the protein surface and the box edge. Counterions (Na^+^ and Cl^–^) were added to neutralize the system and reach a final salt concentration of 150 mM. Energy minimization was carried out using the steepest descent algorithm until the maximum force was below 1000 kJ/mol/nm. Equilibration proceeded in two phases: a 100 ps NVT simulation at 300 K, followed by a 1 ns NPT simulation at 1 bar and 300 K. Temperature and pressure coupling were controlled using the V-rescale thermostat^29^ and the Parrinello–Rahman barostat,^30^ respectively. Covalent bonds involving hydrogen atoms were constrained via the LINCS algorithm,^31^ and long-range electrostatics were treated with the particle mesh Ewald (PME) method.^32^

Production simulations were run for 100 ns under NPT conditions, saving snapshots every 10 ps. Backbone atom deviations and flexibility were analyzed using gmx_rms and gmx_rmsf to calculate root-mean-square deviation (RMSD) and root-mean-square fluctuation (RMSF), respectively.

## Results

### Biochemical and Enzymatic Activity of DsKabA

DsKabA was cloned into the pET-30a vector to produce recombinant proteins with C-terminal 6xHis tags. The proteins were efficiently expressed in soluble form in *E. coli* BL21­(DE3) cells and purified via Ni-NTA affinity chromatography. SDS-PAGE analysis confirmed a protein band at approximately 52 kDa (Figure 1A). Gel filtration chromatography with a calibration curve of protein sizes (Figure 1B) and SEC-MALS analysis (Figure 1C) indicated a molecular mass of approximately 50.48 kDa, closely matching the theoretical monomeric mass (52,752.39 Da) calculated using the Expasy ProtParam web tool.^33^ The far-UV CD spectrum was used to assess the secondary structure content of DsKabA, revealing well-folded properties with dominant helical characteristics, as indicated by minima at 208 and 222 nm (Figure 1D). Analysis using the K2D3 server^34^ predicted approximately 69.47% helical and 6.63% strand conformations.

**Figure 1:**
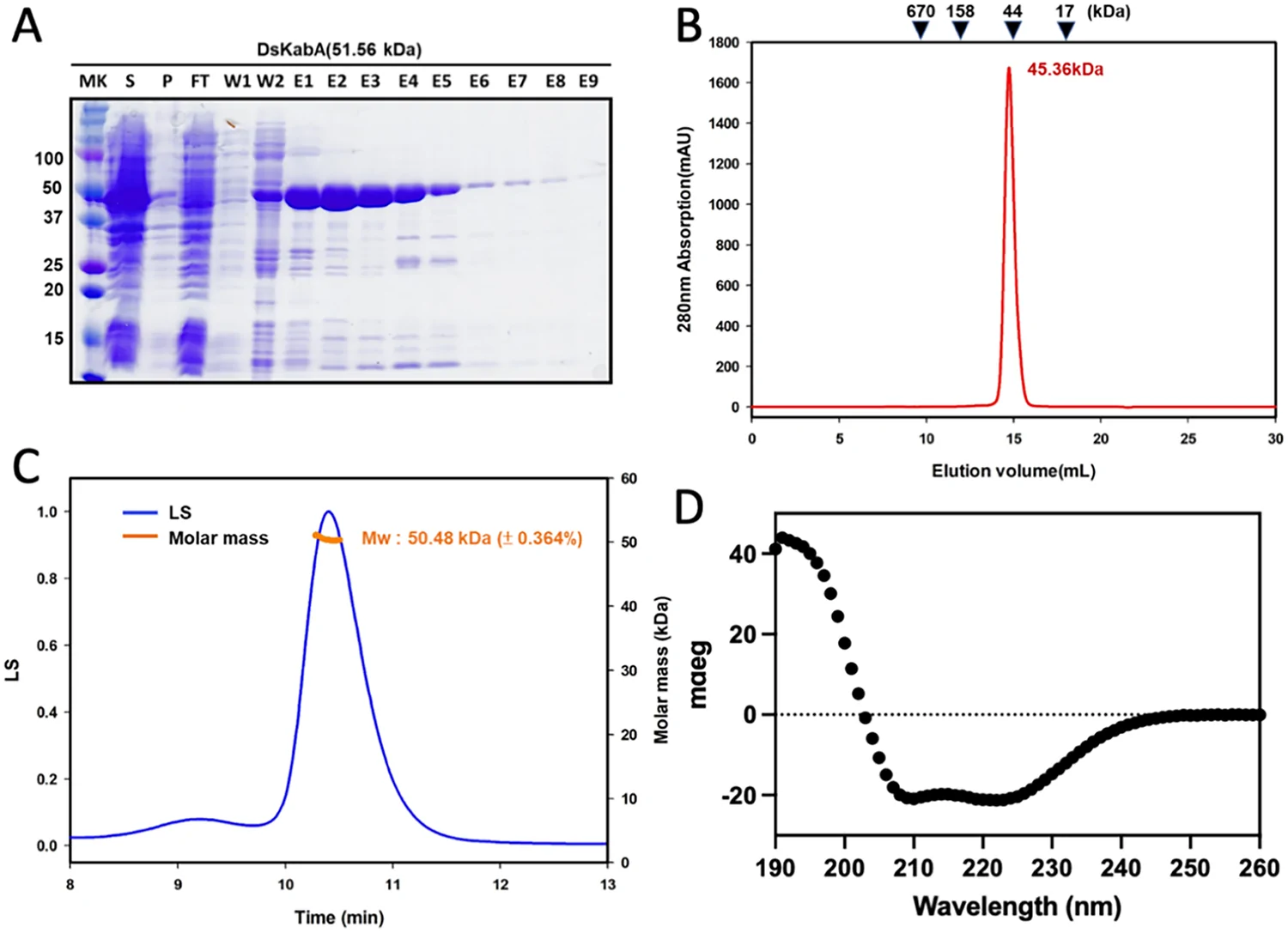
Biochemical characterization of DsKabA. (A) SDS-PAGE analysis of DsKabA purification using a Ni-NTA column with elution buffers of varying imidazole concentrations. MK represents the molecular weight standard; S and P correspond to the supernatant and pellet after cell lysis, respectively; FT indicates the flow-through of the supernatant through the column; W1 and W2 represent washing steps with 0 mM and 25 mM imidazole-Tris buffer; E1–E9 denote elution steps with 50 mM, 75 mM, 100 mM, 300 mM, and 500 mM imidazole-Tris buffer. (B) Size exclusion chromatography (SEC) elution profile of DsKabA, with the black arrow indicating molecular weight markers for reference. (C) Molar mass determination of DsKabA using SEC-MALS. The blue line represents light scattering (LS) at 90 deg, while the orange line indicates the average molar mass measured by MALS, approximately 50.48 kDa. (D) Far-UV circular dichroism (CD) spectra (190–260 nm) of DsKabA, illustrating its secondary structure composition.

### LC-MS Based Validation of DsKabA Product Formation

After purification of recombinant DsKabA, we first examined whether the enzyme catalyzes the formation of PKA from DMAPP and l-Glu. Because this reaction has been reported previously in the original characterization of the kainic acid biosynthetic pathway, this experiment was designed to validate product formation under our assay conditions rather than to establish DsKabA activity de novo.^10^ Chemically synthesized PKA was prepared based on a modified published procedure, purified by HPLC, and confirmed by ^1^H NMR analysis (Figure S1A). The synthetic PKA standard was then used to define the retention time and mass spectrometric signal for product identification.

For enzymatic product validation, DsKabA was incubated with DMAPP, l-Glu, and Mg^2+^, and the reaction mixture was analyzed by LC-MS. A product peak corresponding to synthetic PKA was detected in the DsKabA reaction but was absent or strongly reduced in control reactions lacking enzyme. The identity of the enzymatic product was further supported by its matching retention time and mass signal relative to the synthetic PKA standard (Figure S1B and C). These results confirm that the recombinant DsKabA used in this study catalyzes *N*-prenylation of l-glutamate to form PKA under the LC-MS assay conditions.

When DsKabC was included in the reaction mixture, an additional product peak corresponding to KA was observed by comparison with the commercial KA standard. This result confirms that PKA generated by DsKabA can be further converted by DsKabC in vitro. Together, these product-based analyses verify the functional activity of the purified DsKabA protein and establish the LC-MS workflow used for subsequent kinetic measurements.

### LC-MS-Based Enzyme Activity and Kinetic Analysis

To obtain reliable kinetic parameters based on direct product formation, the catalytic activity of DsKabA was re-evaluated using LC-MS quantification of PKA under initial-rate conditions. Freshly prepared DsKabA was used at a substoichiometric concentration of 25 nM. When DMAPP concentration was varied in the presence of 100 mM l-Glu, the reaction followed Michaelis–Menten behavior, yielding an apparent *K*_m_ of 1.03 ± 0.16 μM. When l-Glu was varied at a fixed DMAPP concentration of 50 μM, an apparent *K*_m_ of 52.25 ± 1.61 mM was obtained (Table S2). Values represent the mean ± SD from three independent kinetic experiments.

These results indicate a substantially lower apparent *K*_m_ for the prenyl donor DMAPP than for the amino acid substrate under the assay conditions used. Because the kinetic parameters were determined by varying one substrate at a fixed concentration of the second substrate, the values are reported as apparent kinetic parameters. Importantly, all kinetic measurements in the revised study were derived from direct quantification of PKA formation rather than from pyrophosphate (PPi) release.

### Overall Structure of DsKabA

After verifying functionality of heterologously expressed DsKabA through kinetic analyses, our subsequent objective was to elucidate the protein’s three-dimensional structure, aiming to understand its specificity and catalytic mechanism. Crystals were successfully obtained by screening and optimizing the conditions for purified DsKabA (Figure S3) and diffracted to 2.85 Å (Table 1). The crystal unit-cell parameter of the DsKabA in apo-form are as follows: *a* = 98.745, *b* = 98.745, *c* = 206.249, with α = 90°, β = 90°, and γ = 90°. These values suggest a tetragonal crystal system with a possible space group of *P*4_1_2_1_2. The data completeness was 99.4%. Using Phaser^35^ in Phenix software suite,^36^ the structure was determined by the molecular replacement method, employing the homologous N-prenyltransferase DabA^7^ (PDB code: 6VKZ) as the searching model. Analysis confirmed that DsKabA belongs to the tetragonal crystal system with the space group *P*4_1_2_1_2, yielding *R*_work_ and *R*_free_ values of 20.35% and 27.37%, respectively (Table 1). The crystal structure of DsKabA consists primarily of α-helices and two short antiparalleled β-sheets. A side view of the structure reveals an oval-shaped architecture, broader at the sides and narrower at the top and bottom. Upon a 90° rotation, the top view shows a central cavity that serves as the catalytic site where substrates bind (Figure 2A). The secondary structure topology comprises 20 α-helices and two sets of antiparallel β-sheets, with both the N-terminus and C-terminus located on the same side (Figure 2B). The dihedral angles (ψ and φ) of the peptide bonds in the crystal structure of DsKabA backbone were analyzed, and a Ramachandran plot was generated to evaluate structural integrity. Approximately 90.4% of the residues were in the most favored regions, 9.4% in additional allowed regions, and one residue each in the generally allowed and disallowed regions (Table 1). This distribution confirms the structural reliability of the DsKabA apo-form crystal.

**Figure 2:**
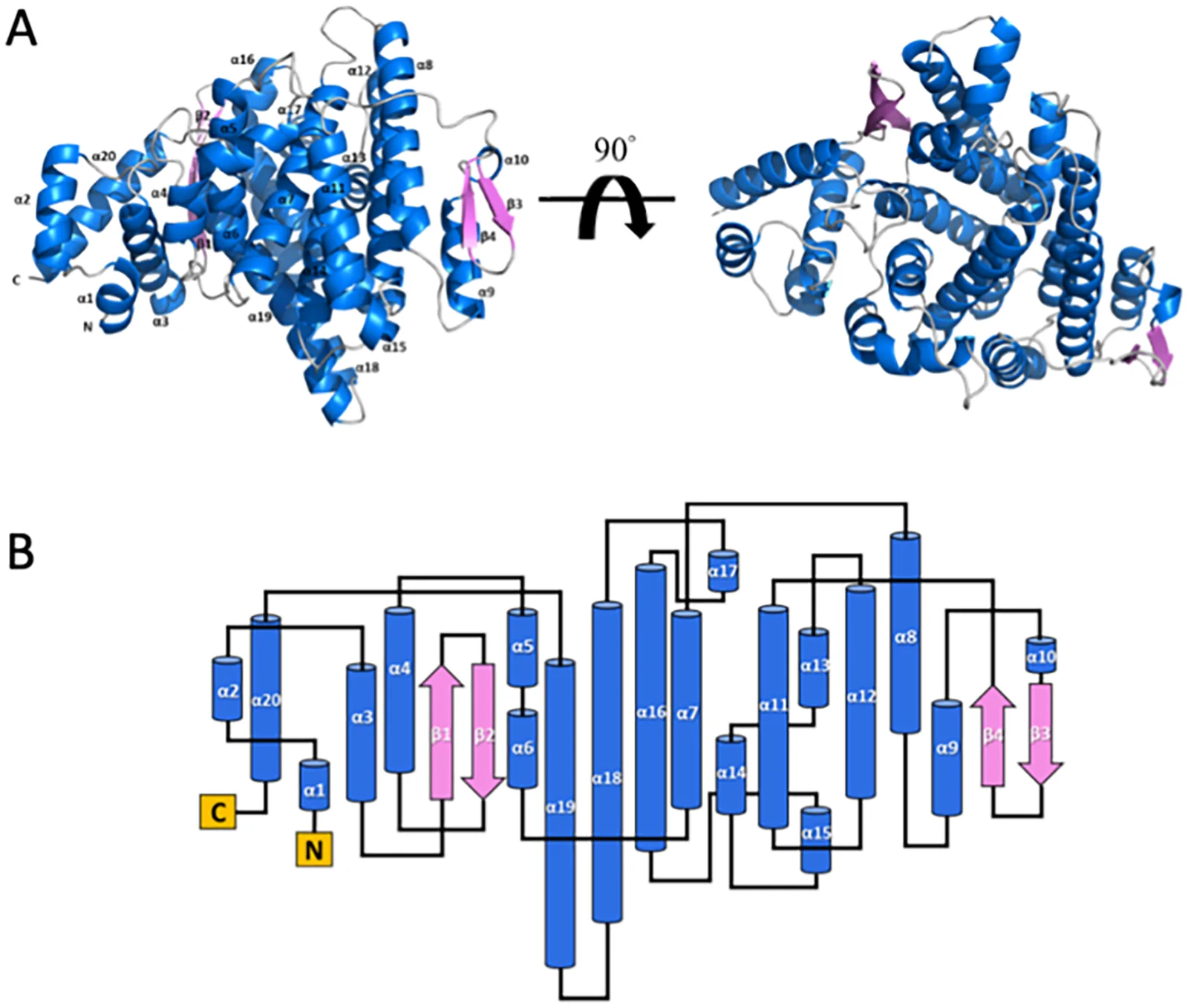
Overall Structure of DsKabA in apo-Form. (A) The overall structure of DsKabA is presented as side and top views in cartoon representation, with α-helices, β-strands, and loops colored in marine, violet, and gray, respectively. (B) A topology diagram illustrating the secondary structure of DsKabA, using the same color scheme as in the cartoon representation in (A).

**Table 1:** X-ray Data Collection and Refinement Statistics of DsKabA

Data collection	DsKabA (PDB code: 9M1W)
Wavelength	1.00000
Resolution range	29.89–2.85 (2.95–2.85)^a^
Space group	*P*4_1_2_1_2
cell dimensions	
*a*, *b*, *c* (Å)	98.745, 98.745, 206.249
α, β, γ	90, 90, 90
Unique reflections	21369 (1402)
redundancy	11.6 (10.7)
Completeness (%)	99.4 (99.9)
Mean I/sigma(I)	13.90 (2.11)
*R*_merge_ ^b^	0.224 (0.654)
CC_1/2_ ^c^	95.9(84.1)
refinement statistics	
*R*_work_ (%)	20.35(26.87)
*R*_free_ (%)	27.37(36.24)
No. of atoms	
Protein	6999
Ligand/Ion	0
Water	0
RMS (bonds)	0.010
RMS angles	1.09
Ramachandran plot	
favored (%)	94.72
allowed (%)	4.92
outliers (%)	0.36
Average B-factor	48.10

a Values in parentheses are for highest-resolution shell.

b *R*_merge_ = ΣhΣi|Ih,*i*-Ih|/ΣhΣiIh,i, where Ih is the mean intensity of the I observations of symmetry related reflections of h.

c CC_1/2_ is the correlation coefficient between intensities from random half-data sets. A derived quantity of CC_1/2_, is an estimate of the “true” CC of the data under examination to the (unknown) true intensities.

Surface and cross-sectional models of DsKabA reveal that its active site is a cavity surrounded by α6, α7, α8, α12, α13, α14, α16, and α19, highlighted in red in the structural models (Figure 3A and 3B). Consurf analysis^37^ was used to assess evolutionary conservation across amino acid positions. Surface models indicate that peripheral residues are largely variable, while residues in the catalytic core exhibit high conservation, suggesting their critical roles in enzymatic catalysis and the catalytic mechanism (Figure 3C). Electrostatic potential mapping shows a predominance of negatively charged residues in the upper catalytic site, likely involved in interactions with Mg^2+^. The lower catalytic site contains a few positively charged residues, potentially responsible for stabilizing the negative charges on pyrophosphate (Figure 3D).

**Figure 3:**
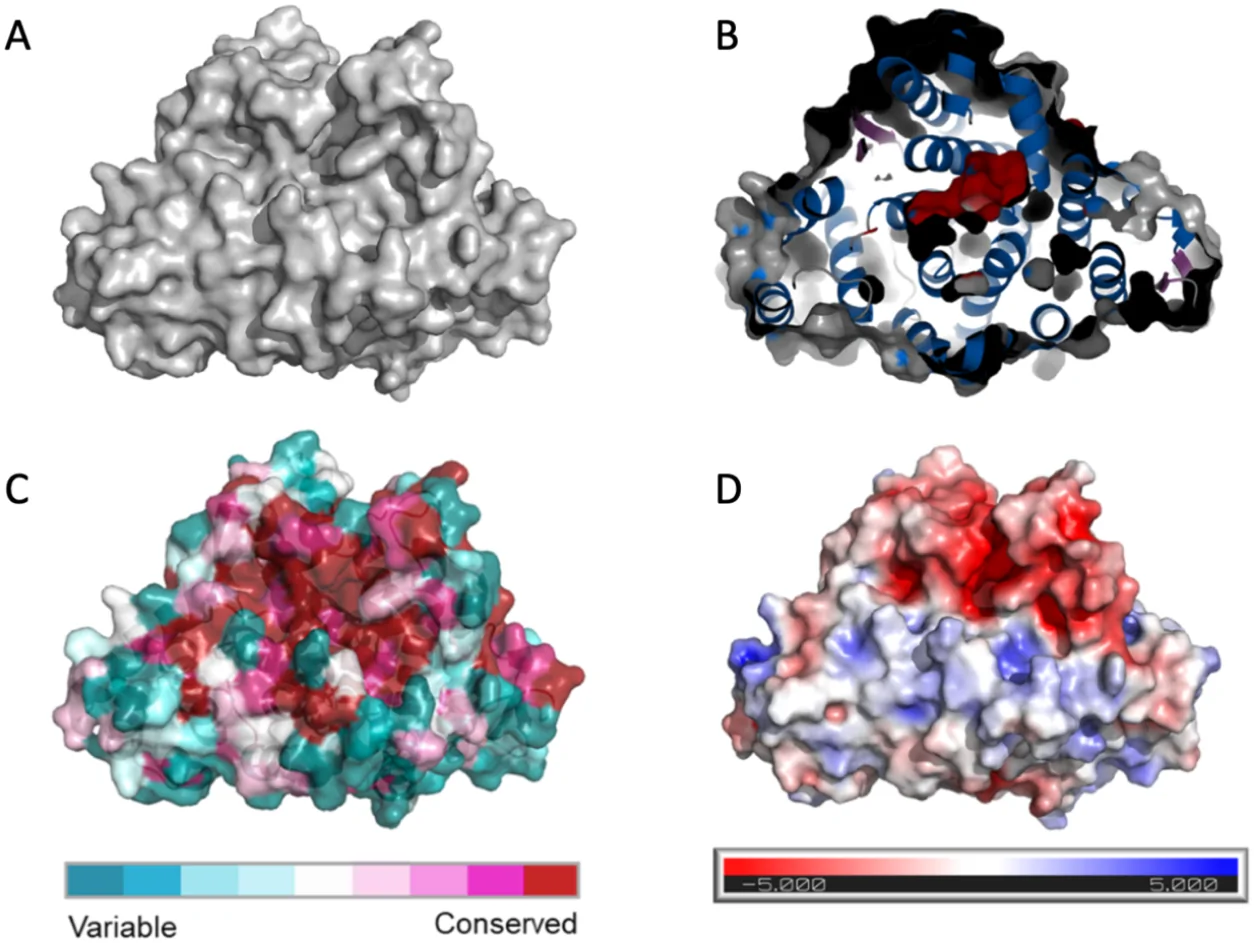
Structural Characterization of DsKabA. (A) The top view of the DsKabA structure is shown as a surface model. (B) A sliced surface view of the DsKabA structure reveals the catalytic cavity, highlighted in red. (C) Evolutionary conservation of amino acid positions in the DsKabA structure is illustrated as a surface model, analyzed using the ConSurf server. Colors correspond to the conservation scale bar. (D) The electrostatic potential of DsKabA is displayed as a surface model, with positive, negative, and neutral regions colored blue, red, and white, respectively, according to the scale bar at the bottom.

Attempts were made to obtain ligand-bound DsKabA structures by both cocrystallization and soaking. Co-crystallization trials were performed in the presence of DMAPP and Mg^2+^, whereas soaking experiments included DMAPP, PPi, and PKA. Most ligand-containing conditions either failed to produce diffraction-quality crystals or resulted in crystal deterioration during soaking. Although crystals were obtained under a DsKabA-PKA-Mg^2+^ condition, the diffraction data were insufficient for reliable ligand modeling. Therefore, a substrate- or product-bound DsKabA structure could not be obtained in the present study. Substrate interactions were instead evaluated using structural comparison with DabA, molecular docking, ITC, SAXS, MD simulations, and structure-guided mutational analysis.

### Molecular Docking of DsKabA with DMAPP

Molecular docking was performed to simulate the interactions between the catalytic center of DsKabA and DMAPP, using the DabA-GSPP-Mg^2+^ structure (PDB code: 6VKZ) as a template. The docking analysis, conducted with Chimera^38^ and AutoDock Vina,^39^ identified potential binding positions of DMAPP and Mg^2+^, along with their interactions with key residues in DsKabA (Figure S4). The predicted binding poses were further refined through energy minimization using the YASARA Energy Minimization Server.^40^

Consistent with previous sequence alignment results, docking analysis confirmed that the conserved NTE motif in DsKabA (N322, T326, E330) plays a crucial role in coordinating Mg^2+^ via side-chain interactions, stabilizing the ion within the active site. Additionally, Mg^2+^ interacts with positively charged residues R417 and R329, facilitating the stabilization of the negatively charged phosphate groups of DMAPP (Figure 4A, 4B and 4C).

**Figure 4:**
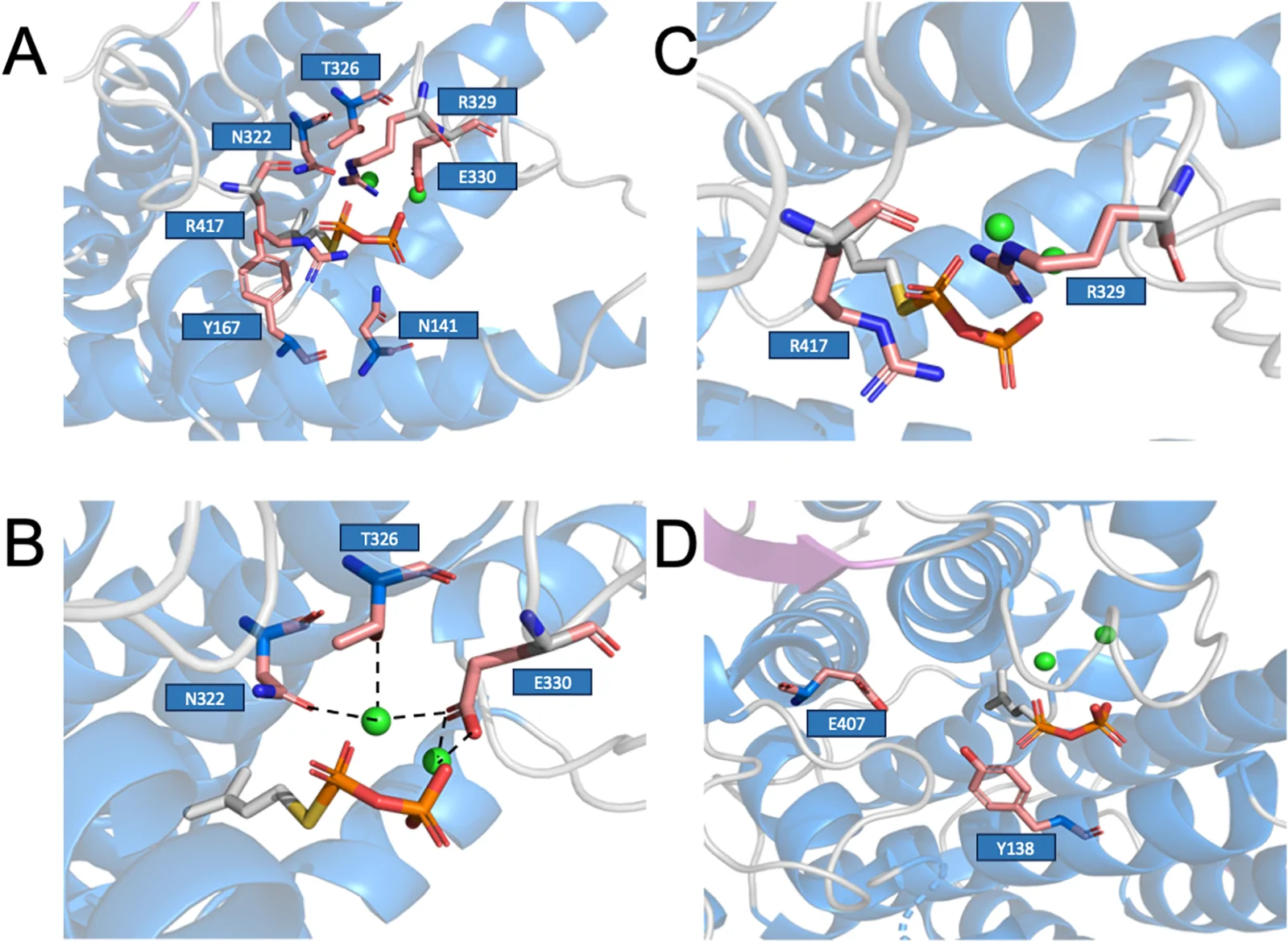
Molecular Docking Analysis of DsKabA Interactions with DMAPP. (A) Active site residues of DsKabA interacting with DMAPP and Mg^2+^. (B) The conserved NTE motif coordinating with Mg^2+^. (C) Two arginine residues stabilizing the phosphate moieties of DMAPP. (D) Positioning of E407 within the reaction cavity. Mg^2+^ ions are shown as green spheres, while key residues and DMAPP are represented as sticks in all panels.

Attempts to dock l-Glu into the active site were unsuccessful. Instead, a cartoon model with a transparent surface representation was used to visualize the substrate binding site and entrance (Figure S4). Given the properties of l-Glu, its backbone amino group exists in the NH_3_^+^ form at pH 8.0 and requires deprotonation to act as a nucleophile. The proximity of E407 to the modeled l-Glu amino group suggests that this residue may contribute to acceptor activation and positioning. This arrangement could facilitate nucleophilic attack by l-Glu on the allylic prenyl donor, accompanied by PPi departure and formation of PKA. (Figure 4D).

### DsKabA Active Site Analysis and Mutagenesis

To experimentally evaluate residues implicated in substrate positioning, catalysis, and prenyl donor recognition, we performed structure-guided mutagenesis of Y403, H406, E407, M114, and M118 (Figure 5A). All purified variants exhibited comparable electrophoretic purity, and far-UV circular dichroism spectra showed similar overall secondary-structure profiles, indicating that the mutations did not cause gross protein misfolding (Figure S5).

**Figure 5:**
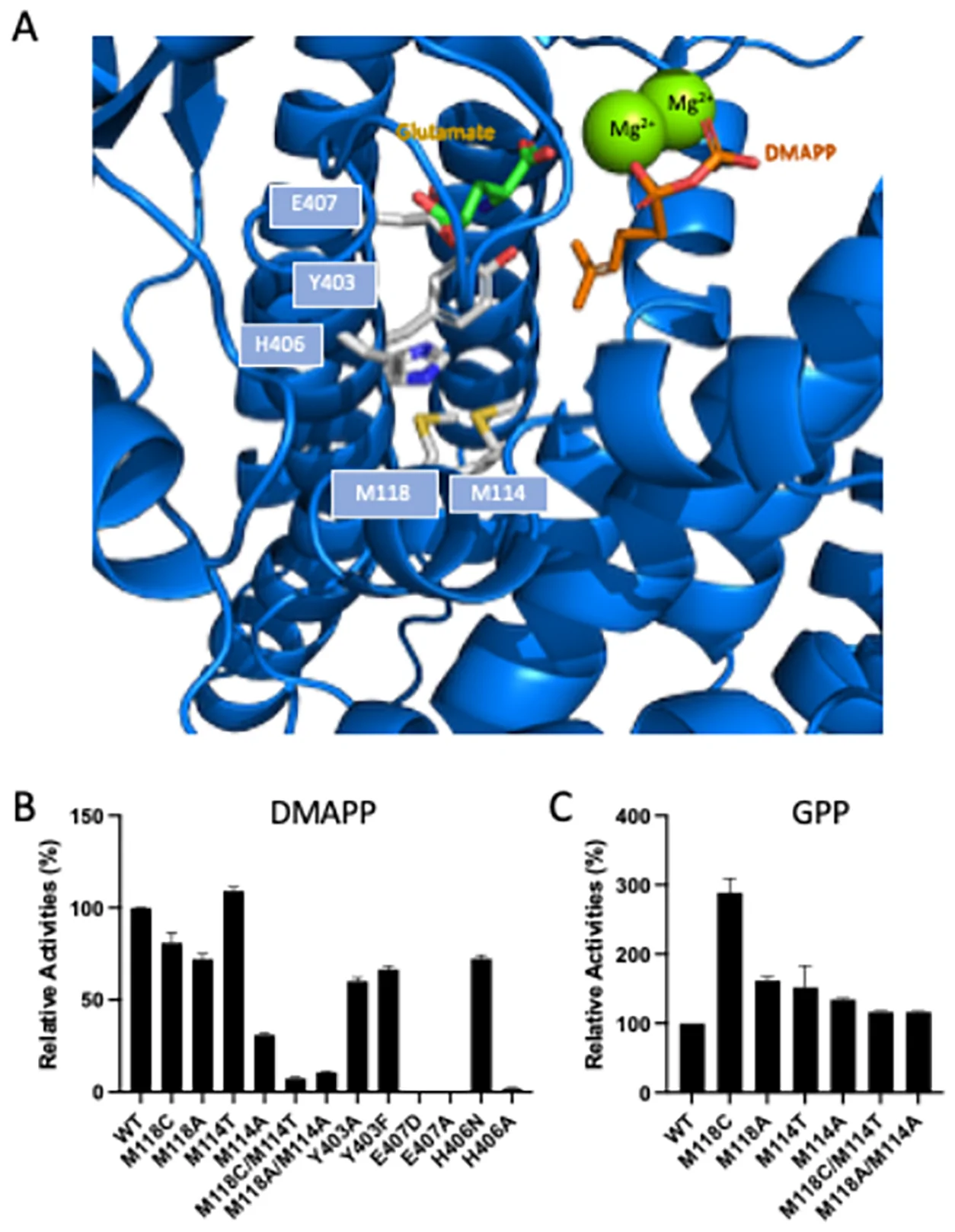
LC-MS based analysis of the relative activities of DsKabA active-site and prenyl donor-binding pocket variants. (A) Locations of Y403, H406, E407, M114, and M118 in the DsKabA active site and prenyl donor-binding pocket. (B) Relative activities of wild-type DsKabA and the indicated variants toward DMAPP, determined by direct LC-MS quantification of PKA formation using the SRM transition *m*/*z* 216.1 → 148.2. (C) Relative activities toward GPP, determined from formation of the corresponding N-geranylated l-glutamate product monitored using the SRM transition *m*/*z* 284.2 → 148.2. Activities were normalized to that of wild-type DsKabA under the corresponding prenyl donor condition, which was defined as 100%. Data represent the mean ± SD of three independent measurements.

Mutations in the Y403–H406-E407 region produced distinct effects on activity with DMAPP. Y403A and Y403F retained approximately 60% and 67% of wild-type activity, respectively, indicating that Y403 contributes to optimal catalytic organization but is not absolutely required for activity. H406N retained approximately 72% activity, whereas H406A showed only ∼2% residual activity, suggesting that the polar side chain at this position is important for productive substrate positioning. In contrast, both E407A and the conservative E407D substitution abolished detectable activity, demonstrating a stringent requirement for the native E407 side chain and supporting an essential role for this residue in catalysis (Figure 5B).

Residues M114 and M118, located near the prenyl donor-binding pocket, showed a different mutational pattern. M118C and M118A retained approximately 81% and 72% of wild-type DMAPP activity, whereas M114T showed activity comparable to or slightly higher than wild type (∼109%). In contrast, M114A retained only ∼31% activity. Notably, the double substitutions M114T/M118C and M114A/M118A reduced DMAPP activity to approximately 7% and 11%, respectively, indicating a strong nonadditive effect when both positions are altered. Together with their altered activities toward GPP, these results suggest that M114 and M118 cooperatively contribute to the geometry and donor preference of the prenyl-binding pocket (Figure 5C). The M114T substitution was previously examined in the related KabA/DabA study;^7^ therefore, we interpret this variant in the context of prior work and further extend the analysis by examining M118 and the M114/M118 double substitutions under the same LC-MS-based relative activity assay.

### Two Distinct Conformations of DsKabA

The apo-form crystal structure of DsKabA revealed two molecules within the asymmetric unit: molecule A (cyan) and molecule B (yellow) (Figure 6A). These molecules exhibited high structural similarity, with two key differences (Figure 6B). First, molecule-B lacked a short N-terminal α-helix. Second, it had a 17-residue deletion (^143^QTAHLKLPEEDIQPELV^159^) spanning parts of the α7 and α8 helices near the catalytic center, leading to a helix-to-loop transition. Notably, Y138 and N141, which reside in the α7 helix of molecule-A but transition to a loop in molecule-B, are involved in substrate binding (Figure 6B and 6C). Symmetry analysis confirmed that these differences were not artifacts of crystal packing, as no interactions with neighboring molecules were observed.

**Figure 6:**
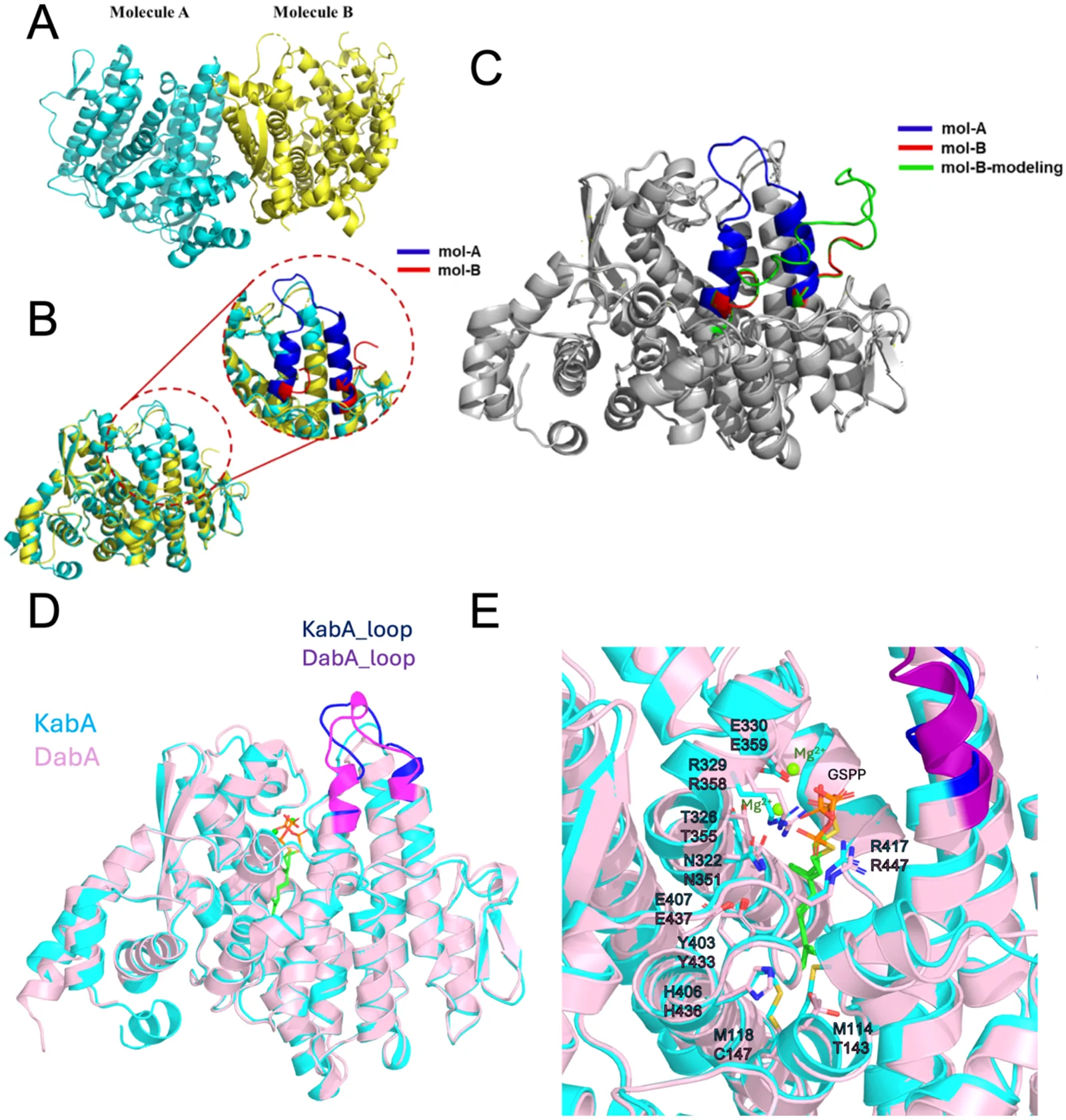
Conformational variation of DsKabA and structural comparison with DabA. (A) The DsKabA crystal structure contains two molecules in the asymmetric unit, molecule-A and molecule-B, colored cyan and yellow, respectively. (B) Structural alignment of molecule-A and molecule-B for comparison. The dashed circle highlights the disordered (missing) α7-loop-α8 segment in molecule-B. The α7-loop-α8 segment of molecule-A and molecule-B are colored blue and red, respectively. (C) The missing α7-loop-α8 segment of molecule-B was modeled and aligned with the structures of molecule-A and molecule-B. The α7-loop-α8 regions of molecule A, molecule B, and molecule B+loop are colored blue, red, and green, respectively, illustrating the different conformations observed or modeled for this region. (D) Structural superposition of apo DsKabA and DabA. DsKabA and DabA are colored cyan and light pink, respectively, whereas their corresponding α7-loop-α8 regions are highlighted in blue and purple. DsKabA and DabA show high structural similarity, with an RMSD of 0.9 Å over 429 aligned Cα atoms. (E) Close-up view of the superimposed active sites of DsKabA and DabA. Residues involved in Mg^2+^ coordination, pyrophosphate stabilization, substrate positioning, and catalysis are shown as sticks. The NTE motif residues N322, T326, and E330 of DsKabA align closely with the corresponding Mg^2+^-binding residues in DabA. R329 and R417, together with Y403, H406, and the proposed catalytic residue E407, occupy positions similar to those of their DabA counterparts. M114 and M118 of DsKabA, corresponding to T143 and C147 of DabA, respectively, are positioned near the prenyl donor-binding pocket and were subsequently examined by mutational analysis.

To further probe the conformational differences between the experimentally determined apo structure and docking-derived ligand-bound model of DsKabA, we performed 100 ns MD simulations of the apo form, the DMAPP-bound complex, and the l-Glu plus DMAPP-bound complex. RMSD analysis revealed that all systems reached equilibrium, with the Glu and DMAPP-bound complex exhibiting slightly higher structural deviations (Figure S6A). RMSF analysis indicated that several loop regions, including those near the active site, were more flexible in the apo state and stabilized upon ligand binding (Figure S6B). Notably, structural superposition showed that the DMAPP-bound complex retained a conformation similar to the apo form (Figure S6C), whereas the l-Glu and DMAPP-bound complex adopted a more closed conformation, particularly in a flexible loop near the entrance of the active site (Figure S6D, highlighted by the red dashed circle). These results suggest that ligand binding promotes ordering of the loop between α7 and α8 helices, potentially stabilizing the active site in a catalytically competent configuration.

To investigate potential conformational differences between the apo and substrate-bound forms of DsKabA, SEC-SAXS analysis was performed. SAXS fitting using CRYSOL in ATSAS software^41^ showed that the monomeric DsKabA crystal structure aligned well with both experimental SAXS profiles (Figure S7A and B). To further explore the functional implications of these conformational differences, the missing α7-loop-α8 segment in molecule-B was modeled, yielding molecule-B+loop. Structural comparison suggested that the structure between α7 and α8 adopts two distinct conformations, corresponding to a closed form (molecule-A) and a putative open form (molecule-B+loop) (Figure 6C).

Additionally, ab initio models generated by DAMMIF^24^ revealed overall shape variations between DsKabA and the DsKabA-DMAPP-Mg^2+^ complex. However, the differences in the α7-loop-α8 region were subtle rather than pronounced (Figure S7C and D). While these findings suggest potential conformational flexibility, current SAXS data alone is hard to confirm whether these conformations represent distinct functional states in solution.

### Structure Comparison of DsKabA with Its Relatives

Structural similarity searches using the Dali Server^42^ identified five proteins with significant resemblance to DsKabA (Z score >28) (Table S3): N-prenyltransferase DabA from *Pseudonitzschia* (PDB code: 6VKZ),^7^ labdane-related diterpenoid synthase LrdC from *Streptomyces sp.* K155 (PDB code: 6OH7),^43^ pentalenene synthase PS from *Streptomyces sp.* UC5319 (PDB code: 6WKI),^44^ geosmin synthase GS from *Streptomyces coelicolor* (PDB code: 5DZ2),^45^ and selina-diene synthase SdS from *Streptomyces pristinaespiralis* (PDB code: 4OKM).^46^ Among these structural relatives, only DabA is classified as an N-prenyltransferase, whereas the remaining four are canonical class I terpene synthases or cyclases. Consistent with their structural relationship, all six proteins share a predominantly α-helical globular architecture with a centrally located active-site cavity. However, DsKabA and DabA contain two additional antiparallel β-sheets on both sides of the structure, resulting in a broader overall conformation compared with the canonical terpene synthase-like enzymes (Figure S8).

Canonical class I terpene synthases typically contain two conserved metal-binding motifs, DDxxxE and NDxxS/TxxxE (NSE/NTE), which coordinate Mg^2+^ ions during catalysis.^47–49^ Sequence alignment showed that DsKabA retains the NTE motif, composed of N322, T326, and E330, whereas the canonical DDxxxE motif is replaced by an NDxxxH sequence (Figure S9). This motif arrangement suggests that DsKabA uses a modified metal-binding architecture relative to canonical class I terpene synthases. Compared with the broader terpene synthase-like relatives, conservation around the catalytic region is limited. In contrast, DsKabA and DabA display strong conservation of the active-site architecture, consistent with their shared function as l-glutamate *N*-prenyltransferases.

Because DabA is the closest structurally and functionally characterized homologue of DsKabA, we performed a focused comparison between the two enzymes. Consistent with the Dali analysis, DsKabA showed the highest structural similarity to DabA, with a Z-score of 60.1, an RMSD of 0.9 Å over 429 aligned Cα atoms, and 67% sequence identity. Superposition of apo DsKabA with DabA revealed a highly conserved terpene cyclase-like fold and close alignment of the catalytic core (Figure 6D). The NTE motif residues of DsKabA, N322, T326, and E330, occupy positions similar to the corresponding Mg^2+^-binding residues in DabA. Likewise, R329 and R417, which are positioned near the pyrophosphate-binding region, and the proposed catalytic residue E407 are structurally conserved between the two enzymes (Figure 6E). Y403 and H406 also occupy positions corresponding to Y433 and H436 of DabA, respectively. These observations indicate that DsKabA and DabA share a highly conserved catalytic architecture for l-glutamate *N*-prenylation.

Despite this overall conservation, local differences are evident near the prenyl donor-binding pocket and active-site entrance. In DsKabA, M114 and M118 correspond to T143 and C147 of DabA, respectively (Figure 6E). The M114/T143 position has previously been implicated in prenyl donor selectivity, whereas the potential contribution of the neighboring M118 position has not been established. Our mutational analysis showed that substitutions at M114 and M118 altered the relative utilization of DMAPP and GPP, supporting a role for this local region in modulating prenyl donor recognition (Figure 5). In addition, the α7-loop-α8 region of DsKabA adopts distinct conformations in the two crystallographic protomers and is located adjacent to the active-site entrance (Figure 6D). Together, these observations indicate that DsKabA retains the conserved catalytic framework of DabA while exhibiting local structural and conformational features that may modulate prenyl donor accommodation and substrate access.

### Substrate Binding and Catalysis Investigated by ITC

Isothermal titration calorimetry (ITC) was employed to investigate the binding interactions between DsKabA and its substrates, DMAPP and l-Glu, as well as Mg^2+^ (Table S4). ITC analysis showed that DMAPP binding was markedly enhanced in the presence of Mg^2+^ (Figure S10A and B). In the absence of Mg^2+^, only weak binding was observed and the titration did not reach complete saturation under the experimental conditions, whereas addition of Mg^2+^ resulted in substantially stronger and more readily quantifiable binding. These results indicate that Mg^2+^ plays a critical role in promoting productive DMAPP association with DsKabA.

For l-Glu, no significant heat changes or binding interactions were observed when it was titrated alone, indicating that detectable binding requires the presence of DMAPP and Mg^2+^ (Figure S10C). To further examine product binding, ITC was performed with PKA in the presence of Mg^2+^. PKA bound to DsKabA with an apparent *K*_d_ of 3.38 μM and was associated with relatively small heat changes (Figure S10D). When DsKabA was preincubated with DMAPP and Mg^2+^, subsequent addition of l-Glu produced measurable heat changes and yielded an apparent *K*_d_ of 0.64 μM. The endothermic binding profile suggests that l-Glu preferentially associates with the preformed DsKabA-DMAPP-Mg^2+^ complex (Figure S10E). Together with the much higher apparent *K*_m_ for l-Glu than for DMAPP, these ITC results are compatible with an ordered binding model in which DMAPP-Mg^2+^ binding precedes and promotes productive l-Glu association. However, additional presteady-state kinetic or variant-binding analyses would be required to fully define the binding sequence.

### Proposed Catalytic Mechanism of DsKabA

Integrating results from enzyme activity assays, molecular docking, and ITC, a catalytic mechanism for DsKabA-mediated *N*-prenylation of DMAPP and l-Glu is proposed (Scheme 2). In the first step, DsKabA coordinates Mg^2+^ via the conserved NTE motif (N322, T326, E330). DMAPP enters the active site, where its phosphate groups are stabilized by R329, R417, and the coordinated Mg^2+^. Subsequently, l-Glu is positioned near E407, placing its amino group in proximity to the allylic prenyl donor. The location of E407 and the strong loss of activity upon mutation are consistent with a role in facilitating acceptor activation and productive substrate positioning. Nucleophilic attack by the amino group of l-Glu on the allylic prenyl donor would then lead to PPi release and formation of PKA. However, the present data do not distinguish between a concerted substitution mechanism and a pathway involving transient allylic cation-like character during PPi departure.

**Scheme 2:**
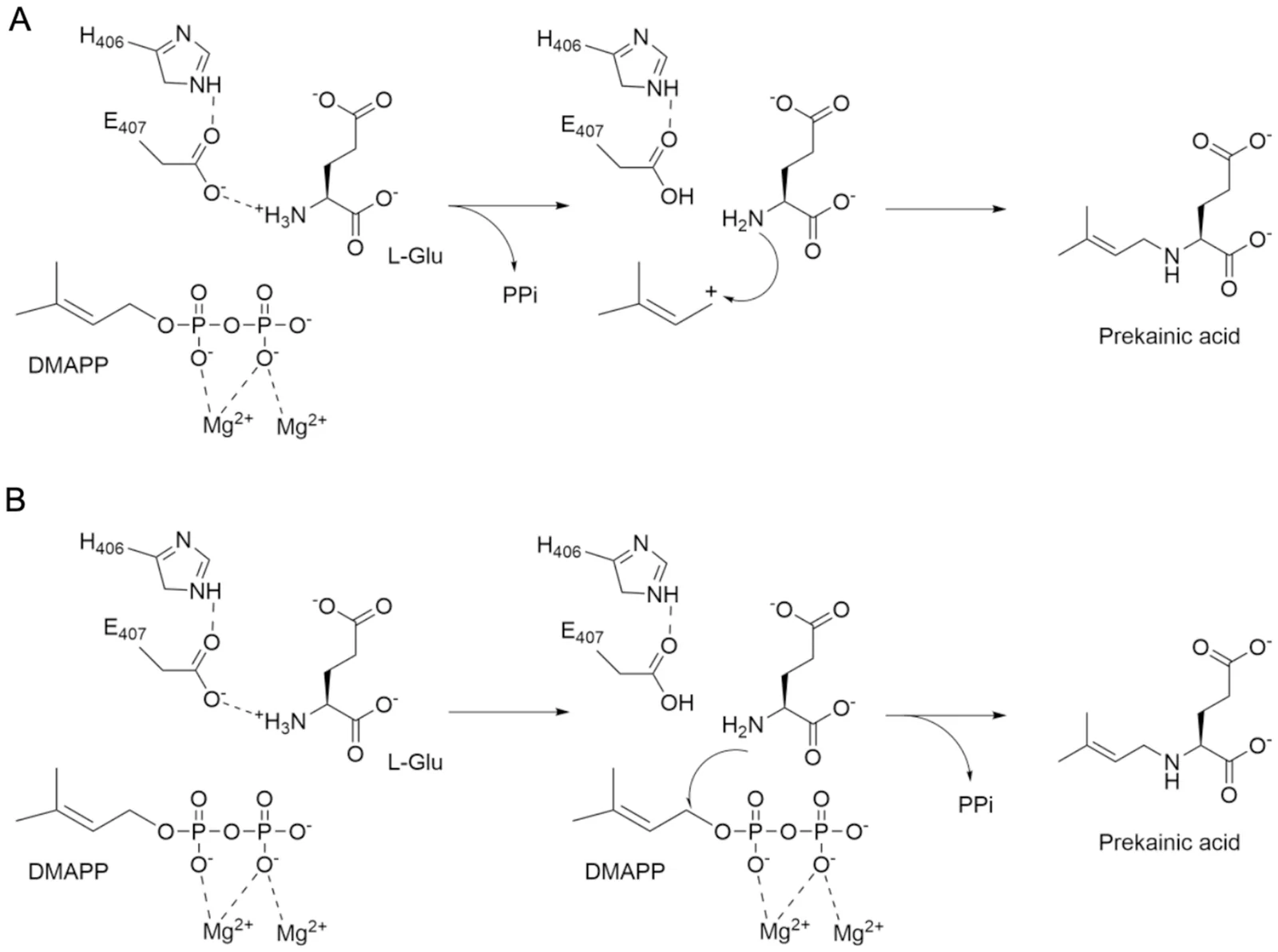
Proposed Catalytic Mechanism for DsKabA-Mediated *N*-Prenylation of l-Glutamate^a^

a (A) In the pathway, binding of DMAPP is stabilized by Mg^2+^ coordination. Ionization and departure of the pyrophosphate group generate a transient dimethylallyl carbocation intermediate. Concurrently, E407, together with H406, may contribute to acceptor activation and productive positioning of l-Glu. The current data are consistent with nucleophilic substitution at the allylic prenyl donor, but they do not distinguish between a concerted pathway and a mechanism involving transient allylic cation-like character during PPi departure. (B) DsKabA first binds DMAPP in a Mg^2+^-dependent manner through the conserved NTE motif. The pyrophosphate group of DMAPP is stabilized by Mg^2+^ and nearby basic residues, including R329 and R417. l-Glu then enters the active site, where E407 is positioned near the amino group and may facilitate its activation for nucleophilic attack on the allylic prenyl donor. This reaction leads to PPi release and formation of PKA. The model is consistent with nucleophilic substitution at the allylic prenyl donor, although transient allylic cation-like character cannot be excluded based on the current data.

Our mutational analysis demonstrated that substitution of E407 significantly impaired the prenylation of l-Glu by DMAPP, while mutating H406 also led to a marked reduction in catalytic activity. These findings are highly consistent with previous structural and biochemical characterization of the homologous enzyme DabA, in which the corresponding residues (E437 and H436, respectively) were demonstrated to be critical for the prenylation reaction.^7^ By combining our mutational data for DsKabA with structural comparisons to DabA, we propose that E407 is critical for productive catalysis, possibly by contributing to acceptor activation and/or active-site organization, with H406 playing a supportive structural or functional role.

## Discussion

The present study provides structural and biochemical insight into DsKabA, the *N*-prenyltransferase that catalyzes the first committed step in kainic acid biosynthesis. Structural comparison indicates that DsKabA is closely related to DabA and shares a conserved terpene cyclase-like architecture for l-glutamate *N*-prenylation. The high structural similarity between DsKabA and DabA, together with the conserved positioning of the NTE motif, pyrophosphate-stabilizing residues, and catalytic glutamate residue, suggests that DsKabA operates within the established mechanistic framework of DabA/KabA-type amino acid prenyltransferases rather than through a distinct catalytic strategy. Nevertheless, the apo structure of DsKabA provides an additional view of the enzyme in the absence of bound substrate or product and reveals local conformational variation near the active-site entrance.

Although substrate-bound crystals of DsKabA were not obtained, the combined structural, binding, and mutational data support a catalytic model in which the prenyl donor is stabilized by Mg^2+^ coordination and nearby basic residues, while l-Glu is positioned for *N*-prenylation within the active site. The NTE motif residues N322, T326, and E330 are positioned to coordinate Mg^2+^, and R329 and R417 are located near the pyrophosphate group of the prenyl donor. E407 is located near the predicted position of the l-Glu amino group and is critical for productive catalysis, consistent with a role in promoting acceptor activation. Together with the effects of Y403 and H406 mutations, these observations support an active-site organization that facilitates productive substrate positioning. The current data are consistent with nucleophilic attack of the l-Glu amino group on the allylic prenyl donor, but they do not exclude transient allylic cation-like character during PPi departure. The available DabA ligand-bound structures also highlight an important limitation of a simple concerted model. In the product-bound DabA structure, the glutamate-derived moiety of the product occupies a region that overlaps with, or is close to, the diphosphate-binding region observed in prenyl-donor analog-bound structures. This arrangement suggests that l-Glu positioning, pyrophosphate departure, and C–N bond formation may not be strictly simultaneous. Therefore, although our docking, ITC, and mutational data support a catalytic model involving Mg^2+^-dependent prenyl donor binding and productive l-Glu positioning, they do not exclude alternative pathways involving transient ion-pair or allylic cation-like character during PPi departure. This interpretation is consistent with the structural comparison between the product-bound and GSPP-bound DabA structures and supports retaining mechanistic alternatives compatible with these states.

A notable feature of DsKabA is the conformational variability of the α7-loop-α8 region near the active-site entrance. The two crystallographic protomers capture different local conformations in this region, and MD simulations suggest that ligand binding may stabilize a more ordered conformation near the entrance of the catalytic pocket. SAXS analysis further supports that DsKabA maintains a compact monomeric architecture in solution, although the resolution of SAXS is insufficient to define the detailed loop conformation. These observations suggest that the α7-loop-α8 region may contribute to substrate access, active-site closure, or product release, providing a possible structural basis for conformational gating in DsKabA.

The mutational analysis further highlights the importance of the prenyl donor-binding pocket entrance in determining donor preference. Residues M114 and M118 are located near this region, and substitutions at these positions altered relative activity toward DMAPP and GPP. These results are consistent with previous work on DabA/KabA-type enzymes showing that local active-site features can influence prenyl donor selectivity. Thus, DsKabA retains the conserved catalytic framework of this enzyme family while displaying local donor-pocket plasticity that may provide a structural basis for engineering altered prenyl donor specificity.

Overall, this work expands the structural understanding of kainic acid biosynthesis by providing the apo-state structure of DsKabA, identifying local conformational features near the active-site entrance, and linking active-site residues to catalysis and donor selectivity. These findings complement previous ligand-bound studies of related enzymes and provide a framework for understanding how amino acid *N*-prenyltransferases combine conserved catalytic architecture with local structural flexibility to control substrate access and prenyl donor specificity.

## Supplementary Material



## Notes

Primers used in this study (Table S1); Kinetic parameters of DsKabA (Table S2); Structural comparison of DsKabA with structural relatives (Table S3); ITC-derived thermodynamic parameters of DsKabA (Table S4); Analytical characterization of synthetic and enzymatically generated PKA and KA (Figure S1); enzymatic activity and kinetic characterization of DsKabA (Figure S2); micrograph of DsKabA protein crystal (Figure S3); molecular docking analysis of DsKabA with DMAPP (Figure S4); SDS PAGE and Circular Dichroism Analysis of wild-type DsKabA and Mutants (Figure S5); molecular dynamics simulations of DsKabA (Figure S6); SAXS analysis of DsKabA (Figure S7); structural comparison of DsKabA with five structurally similar proteins (Figure S8); sequence alignment of DsKabA and five structurally similar proteins (Figure S9); ITC profiles of DsKabA (Figure S10) (PDF)

## Abbreviations

KA: kainic acid
DsKabA: N-prenyltransferase from *Digenea simplex*
Ni-NTA: nickel nitrilotriacetic acid
DMAPP: Dimethylallyl pyrophosphate
GPP: geranyl pyrophosphate
IPTG: isopropyl β-D-1-thiogalactopyranoside
SDS-PAGE: sodium dodecyl sulfate-polyacrylamide gel electrophoresis
PPi: pyrophosphate
PCR: polymerase chain reaction
HPLC: high performance liquid chromatography
FPLC: fast protein liquid chromatography
CD: circular dichroism
SEC-MALS: size exclusion chromatography-multiangle light scattering
SAXS: small-angle X-ray scattering
WT: wild type.

## References

[1] Nadler J. V. (1981). Minireview.
Kainic acid as a tool for the study of temporal lobe epilepsy. Life Sci..

[2] McGeer E. G., McGeer P. L. (1976). Duplication of biochemical changes
of Huntington’s
chorea by intrastriatal injections of glutamic and kainic acids. Nature.

[3] Ramsey U. P., Bird C. J., Shacklock P. F., Laycock M. V., Wright J. L. (1994). Kainic
acid and 1′-hydroxykainic acid from Palmariales. Nat. Toxins.

[4] Werner P., Voigt M., Keinanen K., Wisden W., Seeburg P. H. (1991). Cloning
of a putative high-affinity kainate receptor expressed predominantly
in hippocampal CA3 cells. Nature.

[5] Dingledine R., Borges K., Bowie D., Traynelis S. F. (1999). The glutamate
receptor ion channels. Pharmacol. Rev..

[6] Hampson D. R., Manalo J. L. (1998). The activation of glutamate receptors by kainic acid
and domoic acid. Nat. Toxins.

[7] Chekan J. R., McKinnie S. M. K., Noel J. P., Moore B. S. (2020). Algal neurotoxin
biosynthesis repurposes the terpene cyclase structural fold into an
N-prenyltransferase. Proc. Natl. Acad. Sci.
U. S. A..

[8] Schwob J. E., Fuller T., Price J. L., Olney J. W. (1980). Widespread patterns
of neuronal damage following systemic or intracerebral injections
of kainic acid: a histological study. Neuroscience.

[9] Sperk G. (1994). Kainic acid
seizures in the rat. Prog. Neurobiol..

[10] Chekan J. R., McKinnie S. M. K., Moore M. L., Poplawski S. G., Michael T. P., Moore B. S. (2019). Scalable Biosynthesis of the Seaweed
Neurochemical, Kainic Acid. Angew. Chem., Int.
Ed..

[11] Yazaki K., Sasaki K., Tsurumaru Y. (2009). Prenylation of aromatic compounds,
a key diversification of plant secondary metabolites. Phytochemistry.

[12] Winkelblech J., Fan A., Li S. M. (2015). Prenyltransferases
as key enzymes in primary and secondary
metabolism. Appl. Microbiol. Biotechnol..

[13] Mukai R. (2018). Prenylation
enhances the biological activity of dietary flavonoids by altering
their bioavailability. Biosci Biotechnol Biochem.

[14] Otwinowski Z., Minor W. (1997). Processing of X-ray diffraction data collected in oscillation mode. Methods Enzymol..

[15] McCoy A. J., Grosse-Kunstleve R. W., Adams P. D., Winn M. D., Storoni L. C., Read R. J. (2007). Phaser crystallographic software. J. Appl. Crystallogr..

[16] Adams P. D., Afonine P. V., Bunkoczi G., Chen V. B., Davis I. W., Echols N., Headd J. J., Hung L. W., Kapral G. J., Grosse-Kunstleve R. W. (2010). PHENIX: a comprehensive
Python-based system
for macromolecular structure solution. Acta
Crystallogr., Sect. D:Struct. Biol..

[17] Emsley P., Lohkamp B., Scott W. G., Cowtan K. (2010). Features and
development
of Coot. Acta Crystallogr., Sect. D:Struct.
Biol..

[18] Afonine P. V., Grosse-Kunstleve R. W., Echols N., Headd J. J., Moriarty N. W., Mustyakimov M., Terwilliger T. C., Urzhumtsev A., Zwart P. H., Adams P. D. (2012). Towards
automated crystallographic
structure refinement with phenix.refine. Acta
Crystallogr., Sect. D:Struct. Biol..

[19] Laskowski R. A., MacArthur M. W., Moss D. S., Thornton J. M. (1993). PROCHECK:
a program
to check the stereochemical quality of protein structures. J. Appl. Crystallogr..

[20] Chen V. B., Arendall W. B., Headd J. J., Keedy D. A., Immormino R. M., Kapral G. J., Murray L. W., Richardson J. S., Richardson D. C. (2010). MolProbity: all-atom structure validation for macromolecular
crystallography. Acta Crystallogr. D Biol. Crystallogr..

[21] Petřek M., Otyepka M., Banas P., Kosinova P., Koca J., Damborsky J. (2006). CAVER: a new
tool to explore routes from protein clefts,
pockets and cavities. BMC Bioinf..

[22] Manalastas-Cantos K., Konarev P. V., Hajizadeh N. R., Kikhney A. G., Petoukhov M. V., Molodenskiy D. S., Panjkovich A., Mertens H. D. T., Gruzinov A., Borges C. (2021). ATSAS 3.0: expanded functionality and new tools for
small-angle scattering data analysis. J. Appl.
Crystallogr..

[23] Svergun D., Barberato C., Koch M. H. J. (1995). CRYSOL - a Program
to Evaluate X-ray
Solution Scattering of Biological Macromolecules from Atomic Coordinates. J. Appl. Crystallogr..

[24] Franke D., Svergun D. I. (2009). DAMMIF, a program
for rapid ab-initio shape determination
in small-angle scattering. J. Appl. Crystallogr..

[25] Meng E. C., Goddard T. D., Pettersen E. F., Couch G. S., Pearson Z. J., Morris J. H., Ferrin T. E. (2023). UCSF ChimeraX:
Tools for structure
building and analysis. Protein Sci..

[26] Abraham M. J., Murtola T., Schulz R., Páll S., Smith J. C., Hess B., Lindahl E. (2015). GROMACS: High
performance
molecular simulations through multi-level parallelism from laptops
to supercomputers. SoftwareX.

[27] Huang J., Rauscher S., Nawrocki G., Ran T., Feig M., de Groot B. L., Grubmuller H., MacKerell A. D. (2017). CHARMM36m: an improved force field
for folded and intrinsically
disordered proteins. Nat. Methods.

[28] Boonstra S., Onck P. R., Giessen E. (2016). CHARMM TIP3P
Water Model Suppresses
Peptide Folding by Solvating the Unfolded State. J. Phys. Chem. B.

[29] Bussi G., Donadio D., Parrinello M. (2007). Canonical
sampling through velocity
rescaling. J. Chem. Phys..

[30] Parrinello M., Rahman A. (1981). Polymorphic transitions
in single crystals: A new molecular
dynamics method. J. Appl. Phys..

[31] Hess B., Bekker H., Berendsen H. J. C., Fraaije J. G. E. M. (1997). LINCS: A linear
constraint solver for molecular simulations. J. Comput. Chem..

[32] Harvey M. J., De Fabritiis G. (2009). An Implementation
of the Smooth Particle Mesh Ewald
Method on GPU Hardware. J. Chem. Theory Comput.

[33] Wilkins M.
R., Gasteiger E., Bairoch A., Sanchez J. C., Williams K. L., Appel R. D., Hochstrasser D. F. (1998). Protein identification and analysis
tools in the ExPASy server. Methods Mol. Biol..

[34] Louis-Jeune C., Andrade-Navarro M. A., Perez-Iratxeta C. (2012). Prediction of protein secondary structure
from circular dichroism using theoretically derived spectra. Proteins.

[35] McCoy A. J. (2007). Solving
structures of protein complexes by molecular replacement with Phaser. Acta Crystallogr., Sect. D:Struct. Biol..

[36] Liebschner D., Afonine P. V., Baker M. L., Bunkoczi G., Chen V. B., Croll T. I., Hintze B., Hung L. W., Jain S., McCoy A. J. (2019). Macromolecular
structure determination using
X-rays, neutrons and electrons: recent developments in Phenix. Acta Crystallogr., Sect. D:Struct. Biol..

[37] Ashkenazy H., Abadi S., Martz E., Chay O., Mayrose I., Pupko T., Ben-Tal N. (2016). ConSurf. 2016:
an improved methodology
to estimate and visualize evolutionary conservation in macromolecules. Nucleic Acids Res..

[38] Pettersen E. F., Goddard T. D., Huang C. C., Couch G. S., Greenblatt D. M., Meng E. C., Ferrin T. E. (2004). UCSF Chimera--a
visualization system
for exploratory research and analysis. J. Comput.
Chem..

[39] Eberhardt J., Santos-Martins D., Tillack A. F., Forli S. (2021). AutoDock Vina 1.2.0:
New Docking Methods, Expanded Force Field, and Python Bindings. J. Chem. Inf Model.

[40] Krieger E., Joo K., Lee J., Lee J., Raman S., Thompson J., Tyka M., Baker D., Karplus K. (2009). Improving physical
realism, stereochemistry, and side-chain accuracy in homology modeling:
Four approaches that performed well in CASP8. Proteins.

[41] Franke D., Petoukhov M. V., Konarev P. V., Panjkovich A., Tuukkanen A., Mertens H. D. T., Kikhney A. G., Hajizadeh N. R., Franklin J. M., Jeffries C. M., Svergun D. I. (2017). ATSAS 2.8: a comprehensive
data analysis suite for small-angle scattering from macromolecular
solutions. J. Appl. Crystallogr..

[42] Holm L. (2022). Dali server:
structural unification of protein families. Nucleic Acids Res..

[43] Centeno-Leija S., Tapia-Cabrera S., Guzman-Trampe S., Esquivel B., Esturau-Escofet N., Tierrafria V. H., Rodriguez-Sanoja R., Zarate-Romero A., Stojanoff V., Rudino-Pinera E. (2019). The structure of (E)-biformene
synthase provides insights into the biosynthesis of bacterial bicyclic
labdane-related diterpenoids. J. Struct. Biol..

[44] Matos J. O., Kumar R. P., Ma A. C., Patterson M., Krauss I. J., Oprian D. D. (2020). Mechanism Underlying Anti-Markovnikov
Addition in the Reaction of Pentalenene Synthase. Biochemistry.

[45] Harris G. G., Lombardi P. M., Pemberton T. A., Matsui T., Weiss T. M., Cole K. E., Koksal M., Murphy F. V. t., Vedula L. S., Chou W. K. (2015). Structural
Studies of Geosmin Synthase, a Bifunctional
Sesquiterpene Synthase with alphaalpha Domain Architecture That Catalyzes
a Unique Cyclization-Fragmentation Reaction Sequence. Biochemistry.

[46] Baer P., Rabe P., Fischer K., Citron C. A., Klapschinski T. A., Groll M., Dickschat J. S. (2014). Induced-fit
mechanism in class I
terpene cyclases. Angew. Chem., Int. Ed..

[47] Cane D. E., Kang I. (2000). Aristolochene synthase: purification, molecular cloning, high-level
expression in *Escherichia coli*, and
characterization of the Aspergillus terreus cyclase. Arch. Biochem. Biophys..

[48] Rynkiewicz M. J., Cane D. E., Christianson D. W. (2001). Structure
of trichodiene synthase
from Fusarium sporotrichioides provides mechanistic inferences on
the terpene cyclization cascade. Proc. Natl.
Acad. Sci. U. S. A..

[49] Starks C. M., Back K., Chappell J., Noel J. P. (1997). Structural basis
for cyclic terpene biosynthesis by tobacco 5-epi-aristolochene synthase. Science.

