# Supplementary material for: Structural and Functional Characterization of the N‑Prenyltransferase DsKabA in Algal Neurotoxin Biosynthesis
Source: J Agric Food Chem. 2026 Oct 28;74(44):34989–5002. doi: 10.1021/acs.jafc.6c12021 (PMC13761273; doi:10.1021/acs.jafc.6c12021)
Supplement: Supplementary file 1 [file jf6c12021_si_001.pdf]

Supporting Information

Structural and Functional Characterization of the *N*-Prenyltransferase DsKabA in Algal Neurotoxin Biosynthesis

Wei-Jia Huang[a,b,1], Sheng-Chia Chen[a,c,1], I-Weh Chien[a], Chia-Hsin Lin[b], Jia-Yi Zhou[a], Bo-Yan Guo[b], San-Chi Chang[a,d], Sheng-Wei Lin[b] and Chun-Hua Hsu[a,b,e,f]*

[a]Department of Agricultural Chemistry, National Taiwan University, Taipei 106319, Taiwan

[b]Institute of Biochemical Sciences, National Taiwan University, Taipei 106319, Taiwan

[c]Department of Seafood Science, National Kaohsiung University of Science and Technology, Kaohsiung 811213, Taiwan

[d]Department of Microbiology, Soochow University, Taipei 111002, Taiwan

[e]Genome and Systems Biology Degree Program, National Taiwan University and Academia Sinica, Taipei 106319, Taiwan

[f]Center for Computational and Systems Biology, National Taiwan University, Taipei 106319, Taiwan

[1]These authors contributed equally to this work.

*Corresponding author

Chun-Hua Hsu

Email: andyhsu@ntu.edu.tw; Tel: +886-2-33664468

Table S1. Primers used in this study.

**mutants**	**primer (F)**	**primer (R)**
Y403A	5’-CTGATTGGTGCAACCATTCATGAAATTTG-3’	5’-GCTATCTTCACGGGTTTTAATCATC-3’
Y403F	5’-CTGATTGGTTTTACCATTCATGAAATTTG-3’	5’-GCTATCTTCACGGGTTTTAATCATC-3’
H406A	5’-CTGATTGGTTATACCATTGCAGAAATTTG-3’	5’-GCTATCTTCACGGGTTTTAATCATC-3’
H406N	5’-CTGATTGGTTATACCATTAATGAAATTTG-3’	5’-GCTATCTTCACGGGTTTTAATCATC-3’
E407A	5’-CTGATTGGTTATACCATTCATGCAATTTG-3’	5’-GCTATCTTCACGGGTTTTAATCATC-3’
E407D	5’-CTGATTGGTTATACCATTCATGATATTTG-3’	5’-GCTATCTTCACGGGTTTTAATCATC-3’
M114A	5’-CGAAAAAGCAGGTAGCCTGATGGCACC	5’-CAGTTGATACGGTTATACTTTTTCATATCGG
M114T	5’-CGAAAAAACCGGTAGCCTGATGGCACC-3’	5’-CAGTTGATACGGTTATACTTTTTCATATCGG-3’
M118A	5’-CGAAAAAATGGGTAGCCTGGCAGCACC	5’-CAGTTGATACGGTTATACTTTTTCATATCGG
M118C	5’-CGAAAAAATGGGTAGCCTGTGCGCACC	5’-CAGTTGATACGGTTATACTTTTTCATATCGG
M114A/M118A	5’-CGAAAAAGCAGGTAGCCTGGCAGCACC	5’-CAGTTGATACGGTTATACTTTTTCATATCGG
M114T/M118C	5’-CGAAAAAACCGGTAGCCTGTGCGCACC	5’-CAGTTGATACGGTTATACTTTTTCATATCGG

Table S2. Apparent kinetic parameters and product formation rates of DsKabA under LC-MS assay conditions.

Substrate	apparent V_{max} (nM PKA · min^{-1})	apparent K_m
L-Glu	25.94 ± 0.22	52.25 ± 1.61 mM
DMAPP	35.77 ± 0.87	1.03 ± 0.16 μM

Table S3. Structural comparison of DsKabA with structural relatives using DALI server

PDB code	Protein	Cα atoms	Z-score	RMSD	Identity (%)
6VL1-A	DabA	429	60.1	0.9	67
4OKM-A	SdS	346	30.5	2.4	13
5DZ2-A	ScGS	308	30.0	2.9	13
6WKI-B	PS	307	29.3	2.4	13
6OH7-A	LRD	311	28.6	2.9	12

Table S4. Thermodynamic parameters of DsKabA titrated with substrates and product

Substrate	K_d (μM)	n	ΔH (kJ/mol)	TΔS (kJ/mol)	ΔG (kJ/mol)
DMAPP	>239	0.60	ND	ND	ND
DMAPP+Mg^{2+}	5.53	0.66	-17.13	12.88	-30.01
L-Glu	ND	ND	ND	ND	ND
PKA+Mg^{2+}	3.38	0.69	-4.60	19.21	-23.81
L-Glu + preincubated (DMAPP+Mg^{2+})	0.64	2.24	11.59	46.96	-35.37

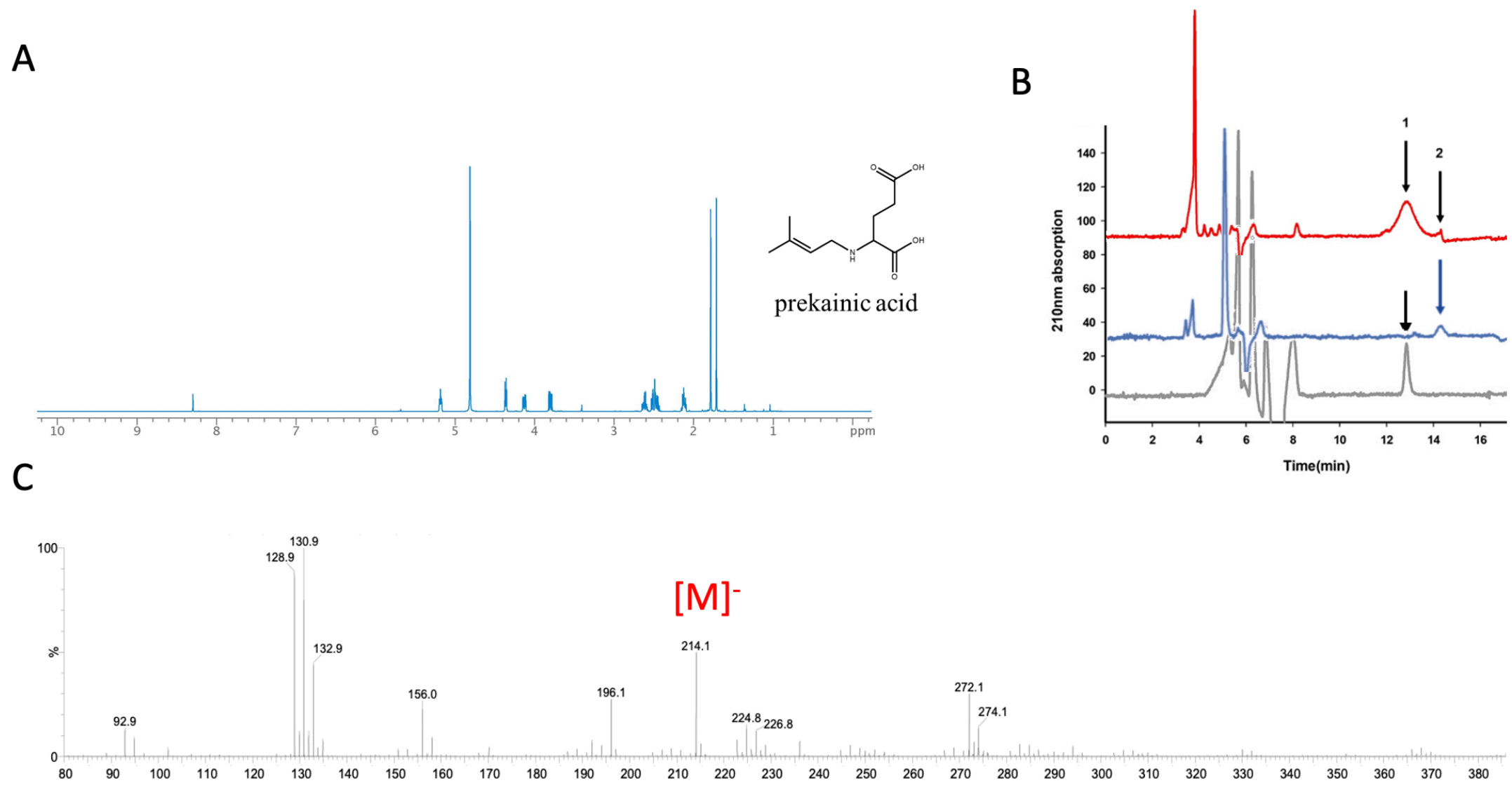

Figure S1. Analytical characterization of synthetic and enzymatically generated PKA and KA.

(A) ^{1}H-NMR spectrum of purified synthetic PKA. (B) HPLC profile of chemically synthesized PKA (black), commercial kainic acid (KA; blue), and the DsKabA/DsKabC enzymatic reaction mixture (red). The indicated peaks correspond to PKA and KA. (C) ESI-MS analysis of the enzymatic product generated by DsKabA, confirming formation of PKA.

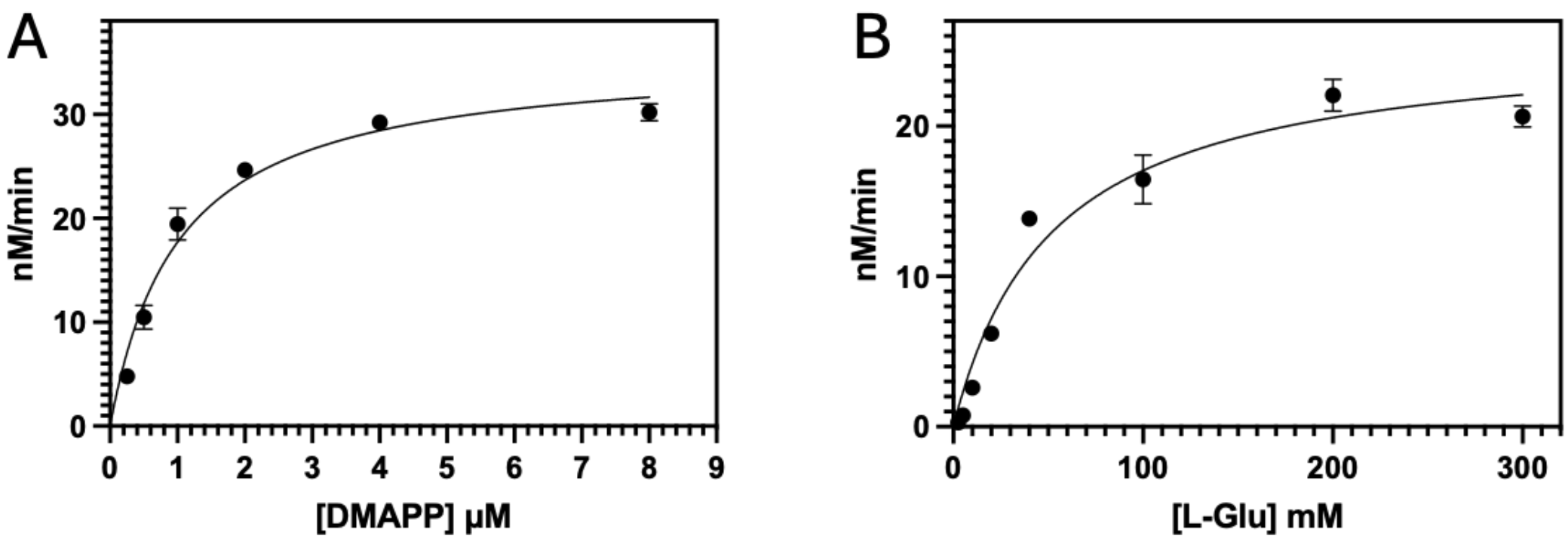

Figure S2. Enzymatic activity and kinetic characterization of DsKabA.

(A) Michaelis-Menten kinetic analysis of DsKabA with DMAPP as the variable substrate in the presence of 100 mM L-Glu. (B) Michaelis-Menten kinetic analysis of DsKabA with L-glutamate as the variable substrate in the presence of 50 µM DMAPP. Enzymatic activity was determined by direct LC-MS quantification of PKA formation. Reactions were performed using 25 nM DsKabA for 15 min. Data represent the mean ± SD from three independent experiments and were fitted to the Michaelis-Menten equation by nonlinear regression.

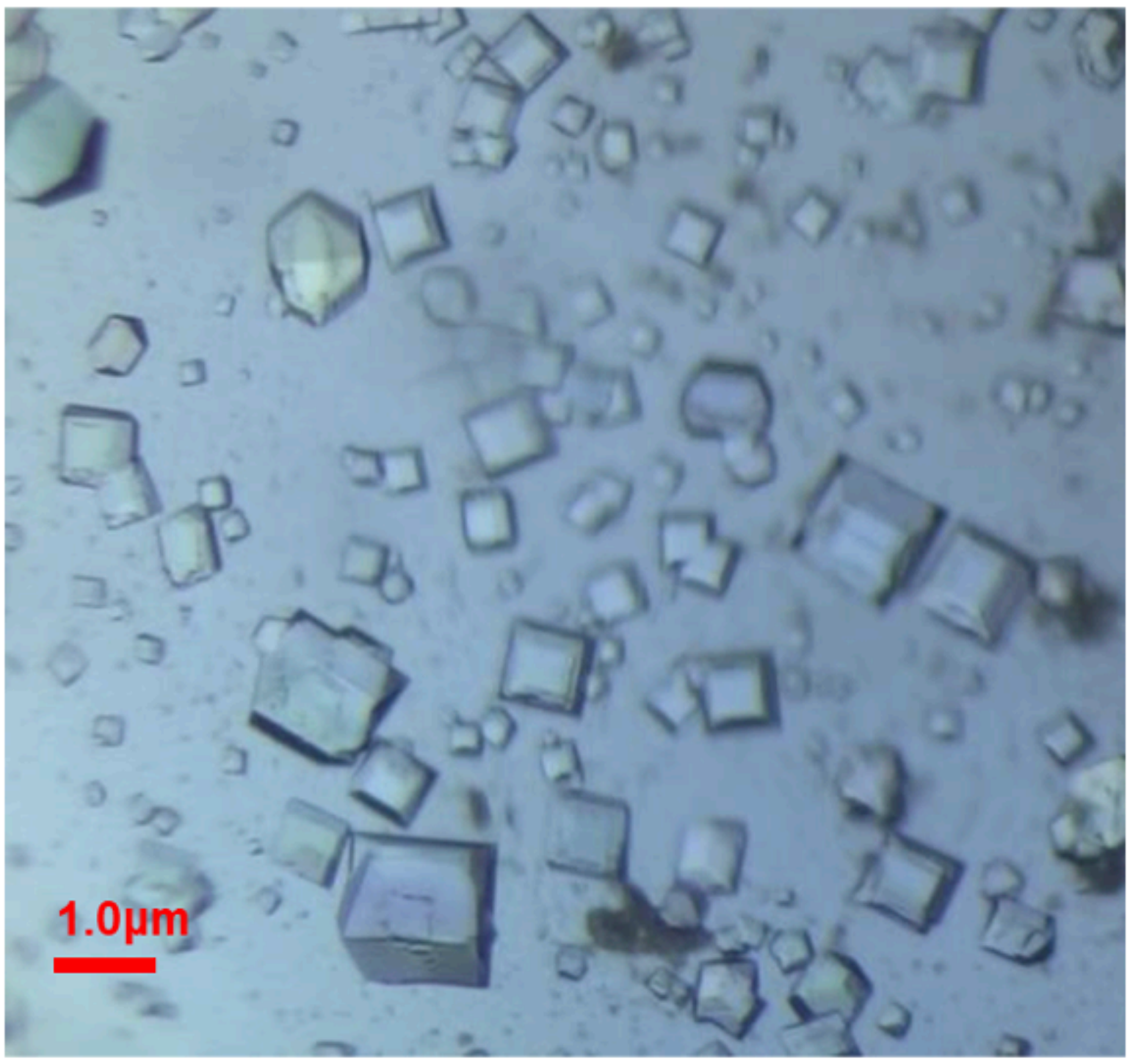

Figure S3. Micrograph of DsKabA protein crystals obtained under optimized crystallization conditions.

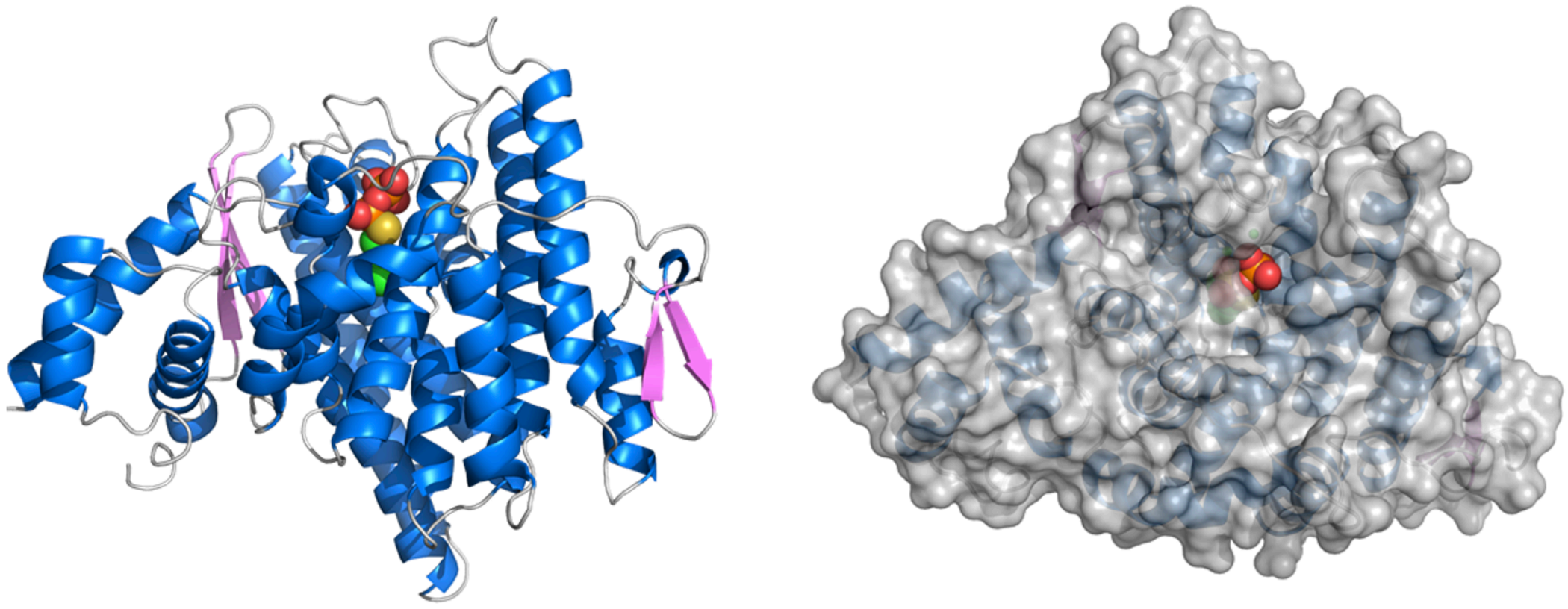

Figure S4. Molecular docking of DsKabA with DMAPP.

Left: The structure of DsKabA bound to DMAPP was simulated using Chimera and AutoDock Vina, with DabA-GSPP (PDB code: 6VKZ) as a template. Right: The potential entry site for DMAPP binding to DsKabA is shown in cartoon and partially transparent surface models. DMAPP is represented as spheres, with carbon, oxygen, and phosphorus atoms colored silver, red, and yellow, respectively.

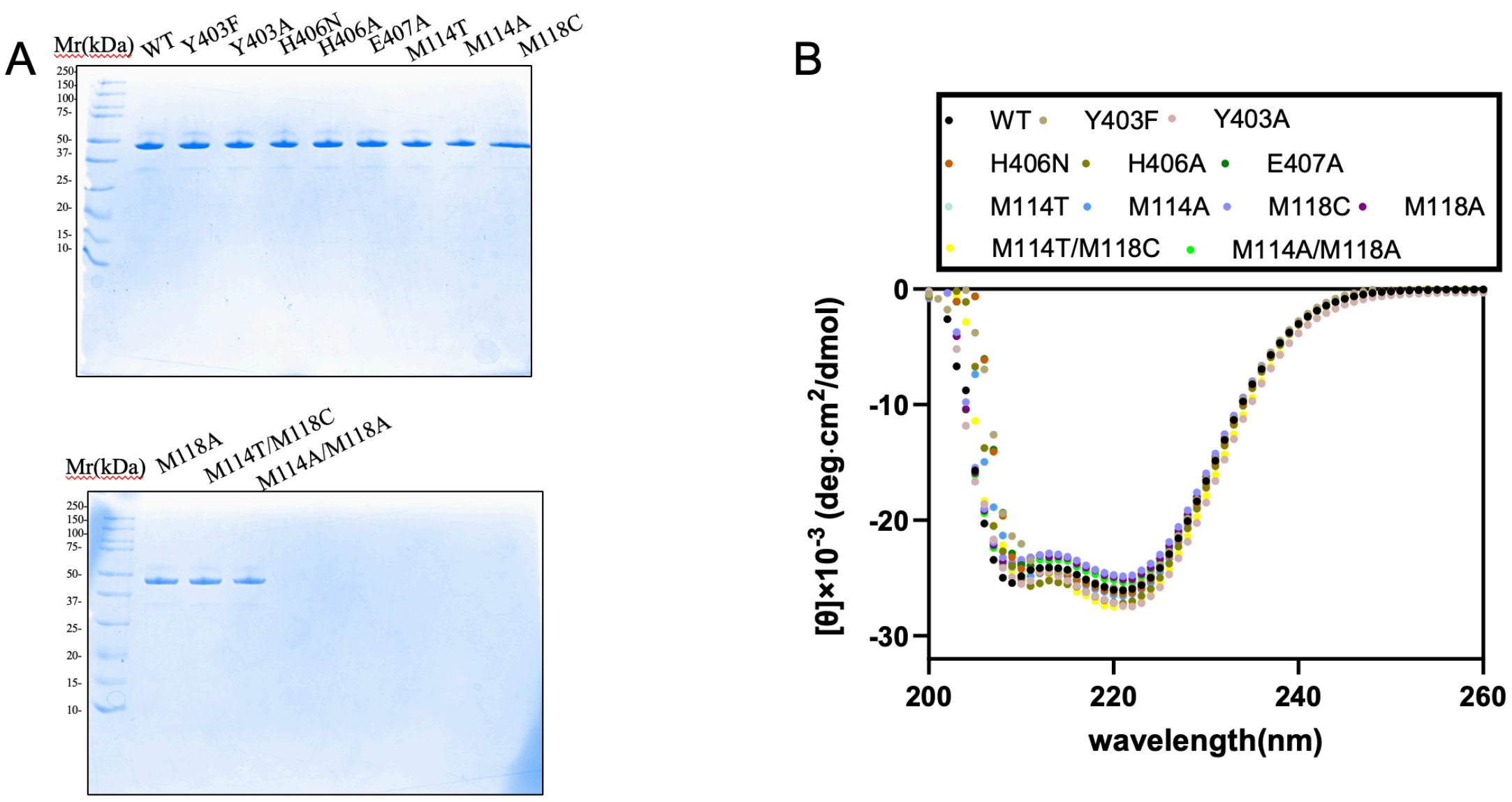

Figure S5. SDS-PAGE and Circular Dichroism Analysis of wild-type DsKabA and Mutants.

(A) SDS-PAGE analysis of purified wild-type and mutant DsKabA proteins. Recombinant enzymes were expressed in *E. coli* and purified by Ni–NTA affinity chromatography. All variants exhibit the expected molecular weight and comparable purity. (B) Far-UV circular dichroism spectra of wild-type and mutant DsKabA proteins measured at 20 °C in phosphate buffer. The similar spectral profiles indicate that all variants retain native-like secondary structure, suggesting that the observed activity differences arise from active-site perturbations rather than global misfolding.

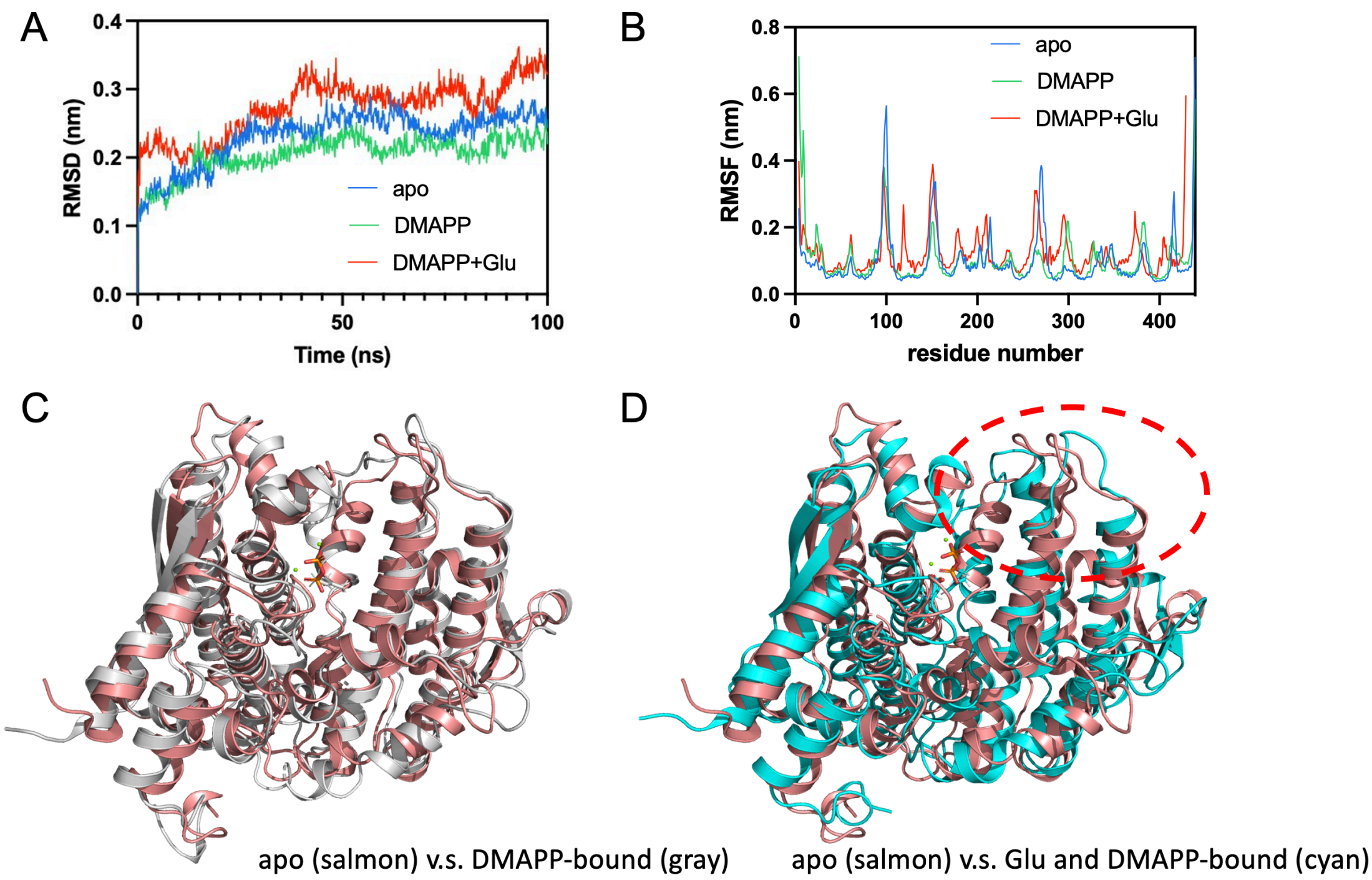

Figure S6. Molecular dynamics simulations of DsKabA in different states.

(A) RMSD profiles of backbone atoms during 100 ns MD simulations of the apo (blue), DMAPP-bound (green), and Glu plus DMAPP-bound (red) states. (B) RMSF analysis showing per-residue backbone fluctuations. Loop regions exhibit decreased flexibility upon ligand binding. (C) Structural alignment of the experimentally determined apo structure (salmon) and docking-derived DMAPP-bound (gray) forms shows minimal overall deviation. (D) Structural superposition of the experimentally determined apo structure (salmon) and docking-derived Glu plus DMAPP-bound (cyan) forms reveals a closed conformation in the ligand-bound state, especially at the flexible loop region near the active site (highlighted by the red dashed circle).

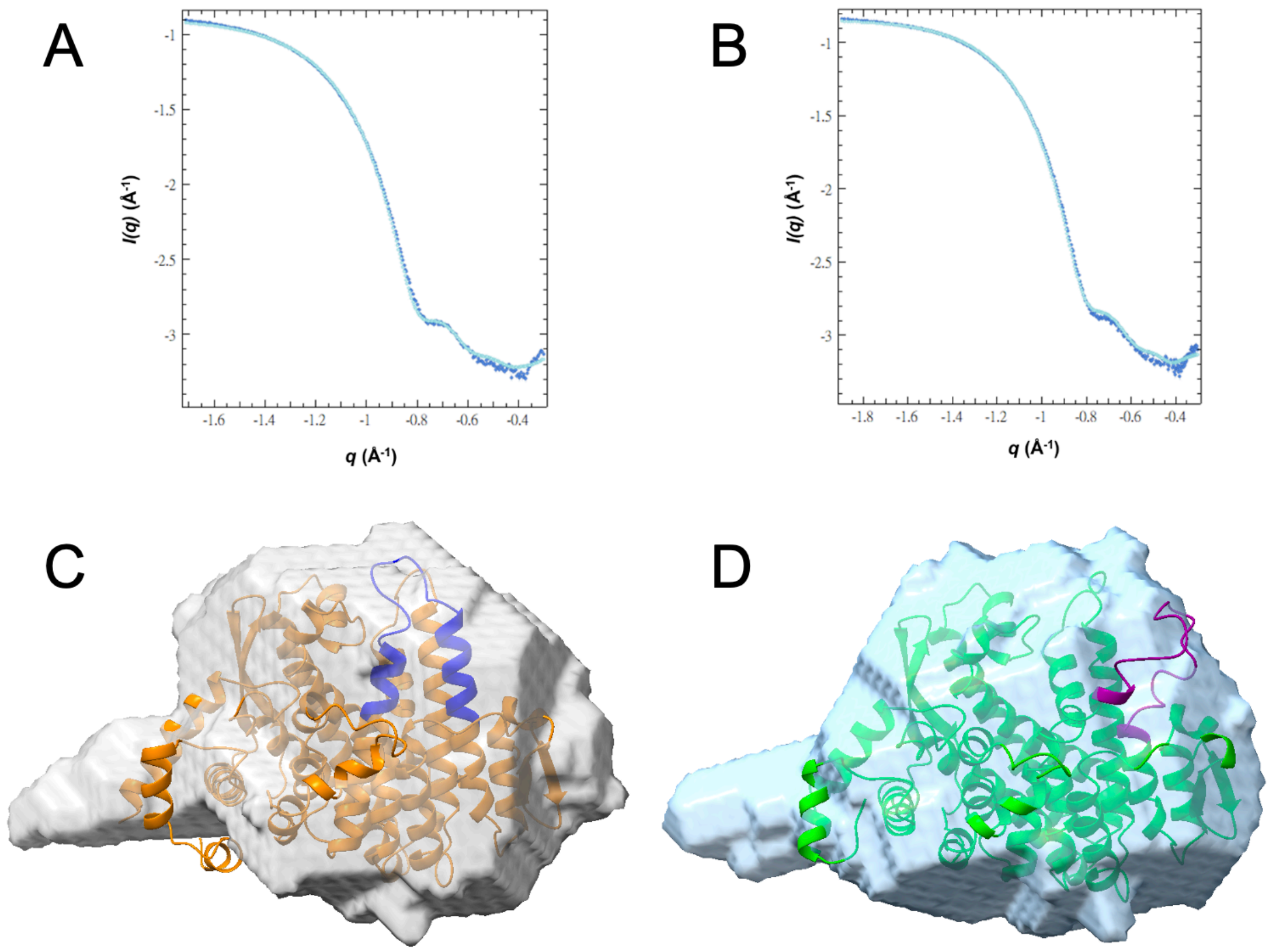

Figure S7. Small-angle X-ray scattering (SAXS) analysis of DsKabA in the presence of DMAPP-Mg^{2+} and in the apo state in solution.

The processed SAXS data were fitted using ATSAS CRYSOL, generating the q versus I(q) profiles. (A) SAXS profile of DsKabA-DMAPP-Mg^{2+} fitted with molecule A, representing a closed, ligand-bound-like conformational model. (B) SAXS profile of apo DsKabA fitted with molecule B+loop, representing an open, apo-like conformational model. Light blue lines represent the SAXS data, whereas dark blue lines indicate the fitting results. (C) and (D) show the corresponding DAMMIF molecular envelopes reconstructed from the data in (A) and (B), respectively. The structures are presented as partially transparent surface models, with molecule A and molecule B+loop shown as cartoon models. These fits support a ligand-associated shift toward a more closed conformation but do not establish experimentally determined substrate-bound and apo-specific solution structures.

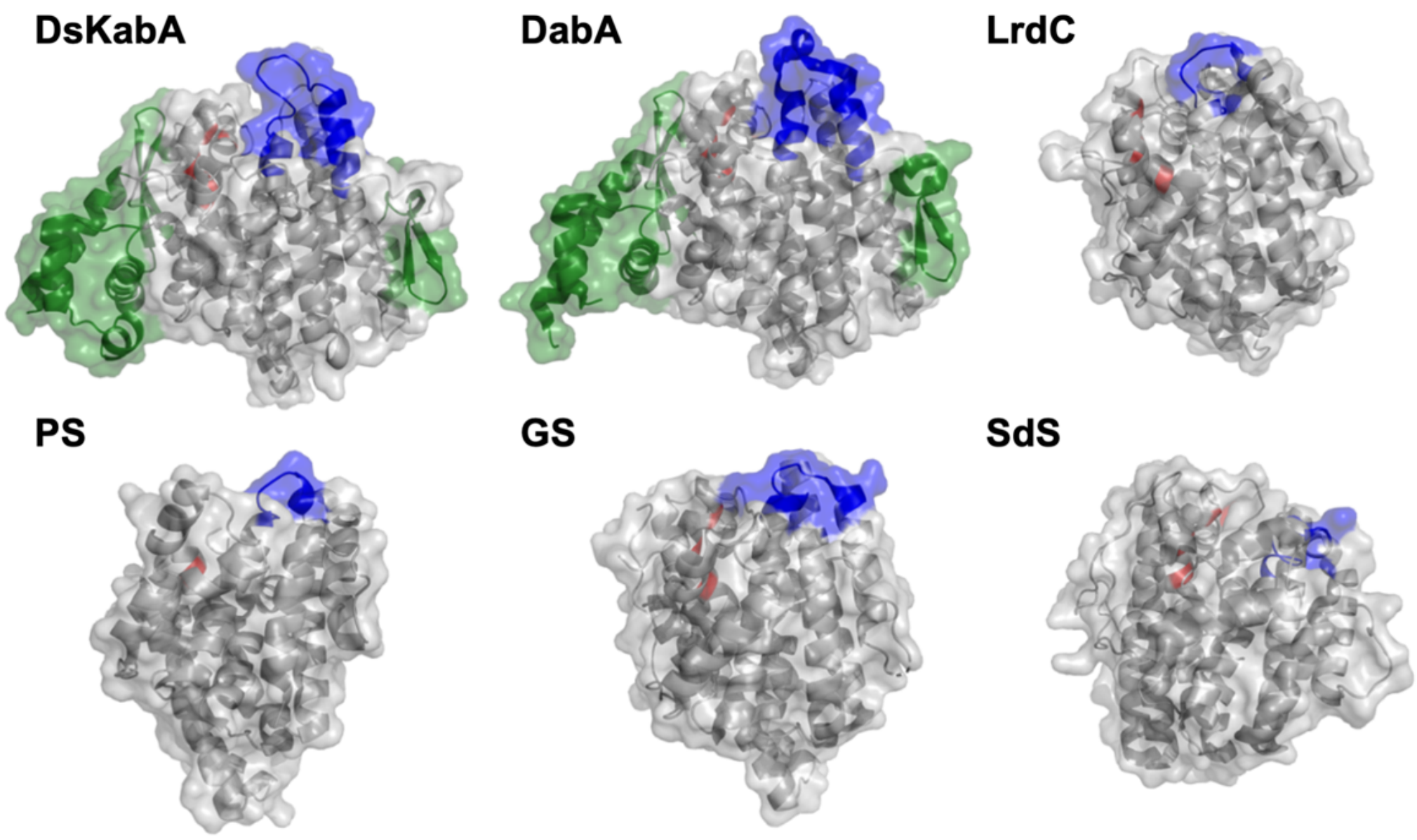

Figure S8. Comparison of the DsKabA structure with five structurally similar proteins identified by the Dali server.

Five structurally similar proteins were identified using the Dali server: DabA (PDB: 6VKZ) from *Pseudo-nitzschia*, LrdC (PDB: 6OH7) from *Streptomyces* sp. K155, PS (PDB: 6WKI) from *Streptomyces* sp. UC5319, GS (PDB: 5DZ2) from *Streptomyces coelicolor*, and SdS (PDB: 4OKM) from *Streptomyces pristinaespiralis*. The structures are shown as cartoon and surface models. The conserved NT/SE motifs are colored red, the extended secondary structures are colored green, and the α7–α8 loop is colored blue.

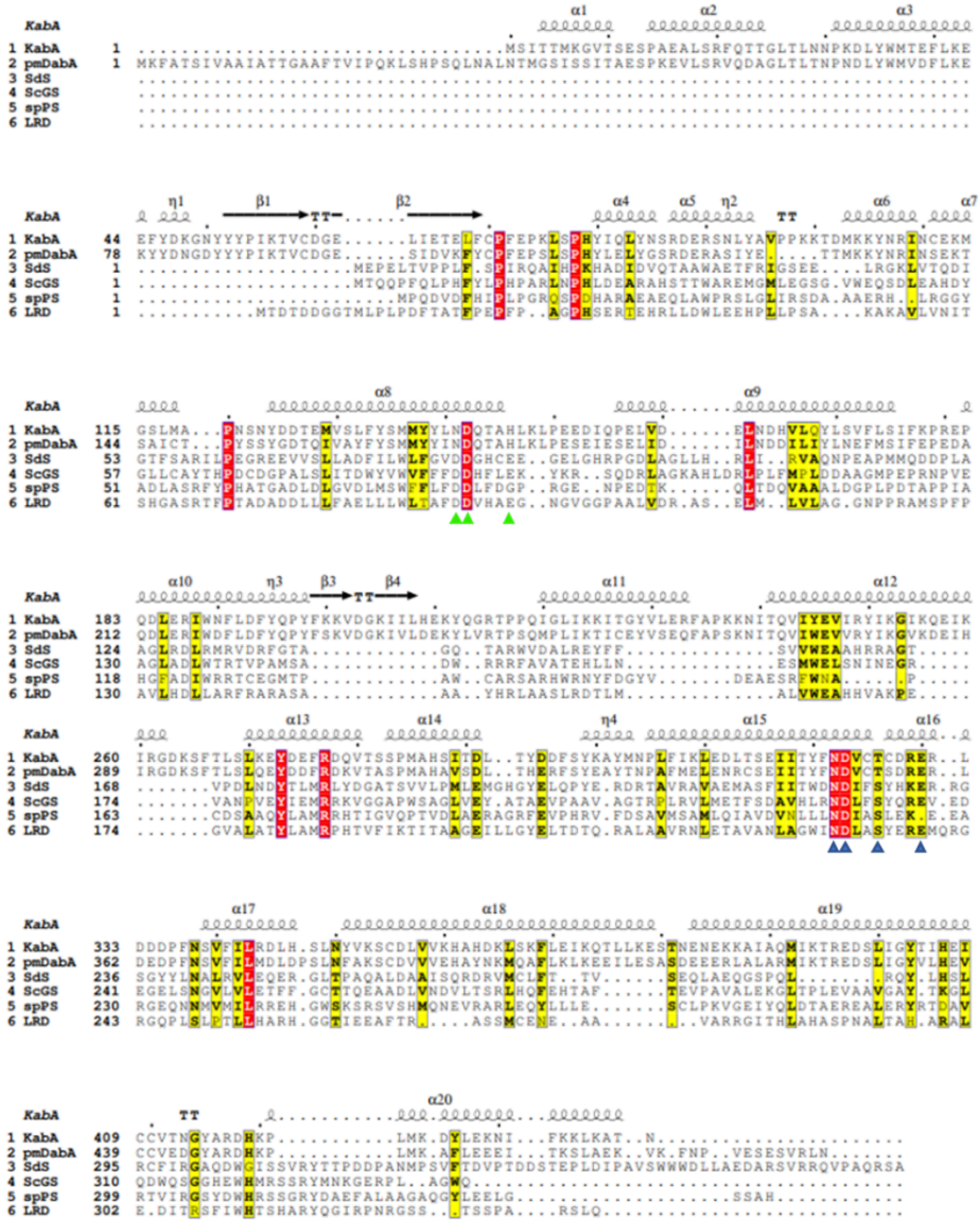

Figure S9. Alignment of DsKabA and five structurally similar proteins identified by the DALI server.

Five structurally similar proteins were aligned to DsKabA using Clustal Omega. The secondary structure elements of DsKabA are shown above the sequence. Blue triangles indicate the NTE/NSE motif, while green triangles indicate the DDxxxE motif. Residues outlined with black boxes represent amino acids with similar physicochemical properties. Residues highlighted in yellow indicate consensus residues with a similarity score > 0.7, and residues highlighted in red indicate identical residues.

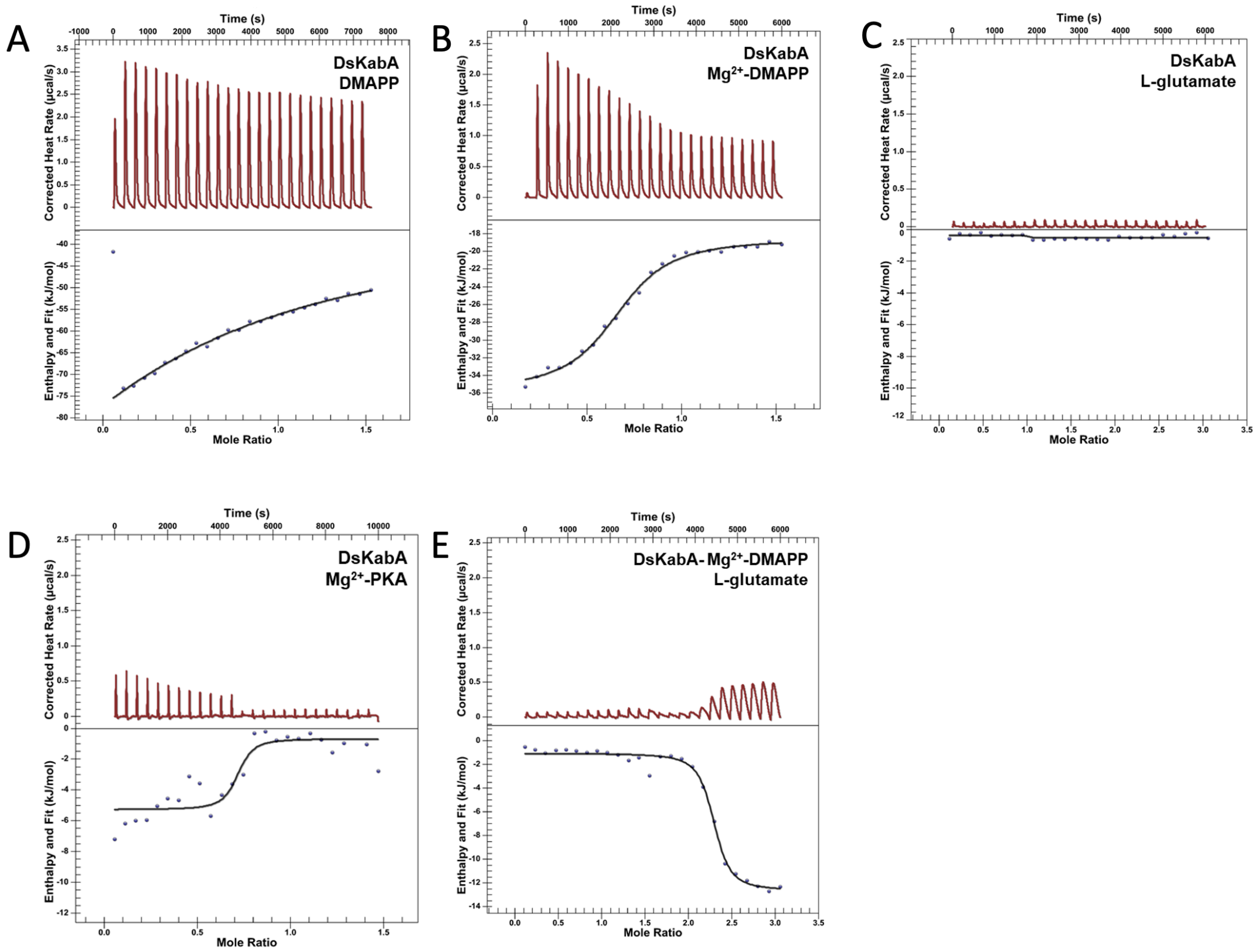

Figure S10. Isothermal titration calorimetry (ITC) profiles of DsKabA titrated with substrates and product.

(A) DsKabA titrated with DMAPP only. (B) DsKabA titrated with DMAPP-Mg^{2+}. (C) DsKabA titrated with L-Glu. (D) DsKabA titrated with prekainic acid (PKA) in solution. (E) Sequential titration of DsKabA, first with L-glutamate, followed by DMAPP. All experiments were performed at 25°C. The top panel shows the corrected heat rate of the titration, while the bottom panel presents the integration of normalized enthalpy per mole versus molar ratio, along with a fitted curve. Thermodynamic parameters from ITC analyses are provided in Table S4.
